# Extracellular vesicle signatures from eye lavage as novel non-invasive biomarkers for hypoxic ischaemic insult—findings from a neonatal mouse model

**DOI:** 10.3389/fmedt.2025.1715676

**Published:** 2025-12-18

**Authors:** Runci Li, Sarah R. Needham, Igor Kraev, Mariya Hristova, Sigrun Lange

**Affiliations:** 1 Department of Neonatology, EGA Institute for Women’s Health, University College London, London, United Kingdom; 2 UKRI: Science & Technology Facilities Council, Central Laser Facility, Rutherford Appleton Laboratory, Oxfordshire, United Kingdom; 3 Electron Microscopy Suite, Faculty of Science, Technology, Engineering and Mathematics, Open University, Milton Keynes, United Kingdom; 4 Pathobiology and Extracellular Vesicles Research Group, School of Life Sciences, University of Westminster, London, United Kingdom

**Keywords:** extracellular vesicles, eye-lavage, hypoxic ischaemic insult, brain, neonate, Gene Ontology, biomarker, early-detection

## Abstract

Neonatal hypoxia ischaemia (HI) affects 1–3 per 1,000 live births, is a major cause of infant mortality and morbidity, and leads to adverse long-term neurological outcomes, while reliable biomarkers are scarce. Extracellular vesicles (EVs) are small membrane vesicles released from cells and play key roles in cellular communication through the transfer of diverse cargoes, including proteins, and can be isolated from various body fluids. Here, we developed a new non-invasive method of biofluid-EV profiling, isolating EVs from eye lavage. Our data demonstrate that in a neonatal HI mouse model of mild and severe insults, significant differences are found in EV eye lavage signatures. We identified increased EV numbers and modifications in EV size profiles and EV's proteomic cargo signatures in eye lavage from HI animals compared to controls. A protein–protein interaction network analysis of the EV proteome cargoes identified enrichment in Gene Ontology and Kyoto Encyclopaedia of Genes and Genomes (KEGG) pathways in the HI groups associated with various homeostatic and disease-related pathways. The specific changes in the mild HI group included pathways for ribosome biogenesis, translation, RNA processing, gene expression, blood coagulation, innate immunity, antioxidant activity, phospholipid binding, post-synapse, cell cortex, and HIF-1 signalling. The enriched pathways only associated with the EV proteome of the severe HI group included cytoskeleton organisation, peptide cross-linking, monosaccharide biosynthesis, peroxidase activity, extrinsic component of plasma membrane, the GAIT complex, mast cell granulation, ruffle, and sealing of the nuclear envelope by the endosomal sorting complex required for transport III. Here, we report a new non-invasive method using eye lavage EV signatures to identify changes in response to HI. Our results highlight eye lavage EVs as potential clinical biomarkers for predicting changes that occur in the brain and eye due to different neonatal HI injury severities.

## 1 Introduction

Extracellular vesicles (EVs) are released from cells and play key roles in cellular communication through the transfer of diverse cargoes, which are comprised of lipids, RNA species, DNA, and proteins. EVs can be isolated from most bodily fluids and are valuable biomarkers with clinical relevance (1). Their use as non-invasive biomarkers for brain-related injury is, therefore, of considerable interest.

Neonatal hypoxia ischaemia (HI) affects 1–3 per 1,000 live births and is a major cause of infant mortality and morbidity. It leads to adverse long-term neurological outcomes, including mental and motor abnormalities, behavioural deficits, developmental delay, learning disabilities, and cerebral palsy (2, 3). In addition, HI can contribute to retinopathy of prematurity, which in its mild form usually heals on its own within 4 months, but severe cases require treatment to prevent complications such as vision loss and retinal detachment (4, 5). As the eye is part of the central nervous system, it can reflect damage occurring to the brain and may offer an approach for detecting specific time windows for early brain-related changes (6). However, limited evidence is currently available regarding the response of the eye to HI damage, including in neonates, and whether the eyes' cellular activation copies the processes observed in the brain. Therefore, it will be of great value to develop new methods, focusing on characterising EV signatures from eye lavage as robust biomarkers for brain and eye pathologies.

Previous research has shown that EVs from various cellular sources have regenerative and neuroprotective potential when applied to different *in vivo* HI models. These include mesenchymal stem cell or mesenchymal stromal cell (MSC)-EVs in a neonatal mouse HI model via intranasal administration (7, 8) and MSC-EVs from human umbilical cord in a neonatal HI rat model (9). The use of EVs as biomarkers for monitoring the downstream effects of HI is less well studied, and the link between eye-EVs and HI has been hitherto understudied. EVs have been isolated from tears in relation to several ocular diseases (10–15), but this is not a feasible approach in neonates, as their tear production is limited. Hence, it is crucial to develop effective methods for eye-fluid-related biomarkers. Protocols for the isolation of EVs from eye lavage have not been published to date, and therefore such an approach is innovative and of potentially high value for clinical application.

Neonatal HI brain injury can be modelled using the modified Rice-Vannucci neonatal mouse model, with unilateral carotid artery ligation, followed by exposure to 8%–10% oxygen for mild (30 min) or moderate/severe (60 min) HI insult. The level of injury is detected histologically using well-established markers, including microglial activation, cell death, and tissue loss post-mortem at 48 h post-injury, and behavioural and histological assessments at later time points (7, 16, 17).

The current treatment for neonatal HI is limited and the technology readiness level (TRL) to assess the damage HI causes to the brain is through neurological signs, Apgar scores, foetal distress, cord acidaemia, and MRI, which are time-consuming, thus delaying diagnosis and treatment (18). Thus, new molecular and cellular biomarkers are urgently needed for the assessment of HI damage to the brain and the eye, where a causal link with HI is hitherto under-investigated.

This study, therefore, aimed to develop a new method for the isolation and characterisation of eye lavage EVs, with a specific focus on neonatal HI models, and identify whether it could be used to reflect brain injury due to different HI severities. Eye lavage EVs and their various cargoes (proteins, RNAs, and lipids) are feasible non-invasive clinical biomarkers with translational potential for a range of neurological and eye-related pathologies.

## 2 Materials and methods

### 2.1 HI mouse model

Neonatal HI brain injury was modelled using the modified Rice–Vannucci neonatal mouse model, with unilateral carotid artery ligation, followed by exposure to 8%–10% oxygen for mild (30 min) or moderate/severe (60 min) HI insult. The level of injury was detected histologically using well-established markers, including microglial activation, cell death, and tissue loss post-mortem at 48 h post-injury, and behavioural and histological assessments at later time points (7, 16, 17, 19).

All animal experiments and care protocols were approved by the United Kingdom (UK) Home Office (PP0028535) and University College London (UCL) Animal Welfare and Ethical Review Board and were carried out according to the United Kingdom Animals (Scientific Procedures) Act 1986. The Animal Research: Reporting of *In Vivo* Experiments (ARRIVE) guidelines were followed. Operations were performed at post-natal day 9 (P9) on C57/BI6 mice (Charles River, United Kingdom) that were bred in-house using a modification of the Rice–Vannucci model of neonatal HI as previously described (7, 16, 17, 19). The parental animals were bred in an environment with a 12 h light/dark cycle and food and water *ad libitum*. The HI procedure was carried out as follows: P9 mice (males and females) were anesthetised using isoflurane (5% induction, 1.5% maintenance). Permanent occlusion of the left common carotid artery was established with 8/0 polypropylene sutures, followed by wound closure with tissue glue. The pups were recovered at 36 °C and then were returned to the dam for 2 h, whereafter they were placed in a 36 °C hypoxic chamber for either 30 min (mild HI) or 60 min (severe HI), with humidified 10% oxygen/90% nitrogen at 3 L/min. The pups were returned to the dam and left for 48 h before eye washing for EV isolation, followed by a standard behavioural assessment and subsequent histological analysis of the extracted brains. All animals (both males and females) from all litters were used in the experiments. Following the HI protocol, the mothers and pups were observed and scored for the welfare of neonatal rodents (20). All pups were taken care of by their mother and achieved overall scores of 0–1 (a score of 0–13 was given for the neonate and a score of 0–6 for mothering ability, where 0 corresponded to normal and 13 and 6 to abnormal behaviour, respectively). Naïve animals served as controls, in addition to sham operation animals (exposed to incision and wound closure, but not to carotid artery occlusion or hypoxia).

### 2.2 Histological analysis of brain tissue for verification of HI

For the histological assessment to determine the extent of HI injury (mild or severe), animals were sacrificed 48 h post-HI insult (30 min or 60 min hypoxia), with an intraperitoneal injection of pentobarbitone and perfusion with 30 mL of 4% paraformaldehyde in phosphate-buffered saline (PBS). Immediately after collection of the eye lavage as described in Section 2.3, the brains were extracted and fixed in 4% paraformaldehyde in PBS at 4 °C for 1 h, followed by cryoprotection in 30% sucrose dissolved in phosphate buffer (PB) for 24 h. Thereafter, the brains were frozen on dry ice, sectioned by cryostat into sequential 40 μm sections, and stored at −80 °C until histological analysis for microglial activation, cell death, and infarct size, with tissue sections scored blindly by two independent observers according to previously published methods (7, 17, 19).

### 2.3 Isolation and characterisation of EVs from eye lavage

For isolation of EVs, the pups' eyes were washed with Dulbecco’s PBS (DPBS) three times, using 20 µL per eye from the sham, naïve, 30-min, and 60-min HI groups, respectively. For the HI animals, eye lavage was collected separately from both eyes, i.e., the eye on the side unaffected by HI damage (but still exposed to hypoxia) and the eye on the affected side (exposed to HI and hypoxia). Eye lavage was pooled for 10 individual eyes per experimental group.

The eye lavage EVs were thereafter isolated using stepwise differential centrifugation, starting at 4,000 g at 4 °C for 20 min, to separate aggregates and apoptotic bodies. The supernatants were collected and moved to new Eppendorf tubes and centrifuged at 30,000*g* for 1 h at 4 °C. The supernatants were discarded and the EV-enriched pellet was then gently washed with 200 µL of DPBS by pipette. The EVs were then centrifuged again at 30,000*g* for 1 h at 4 °C. The supernatants were discarded, and the final EV-enriched pellets were resuspended in 50 µL of DPBS before further analyses, namely, nanoparticle tracking analysis (NTA) for size profiling (Section 2.3.1), transmission electron microscopy (TEM) for morphology analysis (Section 2.3.2), and EV surface marker detection by dot blotting and Western blotting (WB; Section 2.3.3). In addition, the EVs were imaged by direct stochastic optical reconstruction microscopy (dSTORM) for pan-EV and tetraspanin trio EV-markers at the single molecule level (Section 2.3.4). For proteomic cargo analysis, EVs from the sham, naïve, and HI groups were analysed by liquid chromatography-mass spectrometry (LC-MS/MS) and the proteomic content was compared (Section 2.4).

#### 2.3.1 Nanoparticle tracking analysis

The size distribution of the EVs and EV counts were measured by NTA using an NS300 Nanosight (Malvern Panalytical Ltd, Malvern, UK) equipped with a 488 nm blue laser, with the automated syringe pump speed set at 50. The samples were diluted 1/100 (10 µL of EV sample in 990 µL of DPBS) and five 60-s readings were recorded and averaged per sample. The camera settings were set to level 11 for capture, and the post-analysis threshold was set at level 5. Replicate histograms were generated by the NTA software (version 3.0; Malvern Panalytical, UK), showing the median and standard error.

#### 2.3.2 EV surface marker detection by Western blotting

Eye lavage EVs were assessed for two standard EV surface markers, CD63 (ab216130, Abcam, UK) and flotillin-1 (ab41927), by dot blot and Western blot analyses. Before separation on sodium dodecyl sulfate–polyacrylamide gel electrophoresis (SDS-PAGE) 4%–20% Tris-Glycine eXtended (TGX) gels (BioRad, Watford, UK), the EV samples were reconstituted 1:1 in 2 × Laemmli sample buffer and boiled at 100 °C for 5 min. Proteins were transferred onto 0.45 μm nitrocellulose membranes (BioRad, UK) using semi-dry transfer for 1 h at 15 V. For dot blotting, 10 µL of EV solution was dotted onto nitrocellulose membranes using a 10-µL pipette and allowed to dry for 20 min. Following Western or dot blotting, the membranes were blocked for 1 h at room temperature (RT) in 5% bovine serum albumin (BSA, Sigma-Aldrich) in Tris-buffered saline containing 0.1% Tween 20 (TBS-T). Primary antibody incubation was performed overnight at 4 °C on a shaking platform, diluting the primary antibodies at 1/1,000 in TBS-T. The membranes were washed three times in TBS-T and incubated at RT for 1 h in secondary antibody [anti-rabbit IgG horseradish peroxidase (HRP)-conjugated; BioRad, Watford, UK] diluted 1/3,000 in TBS-T. The membranes were washed five times for 10 min in TBS-T and then visualised using a UVP BioDoc-ITTM System (Cambridge, UK) in conjunction with enhanced chemiluminescence (ECL) (Amersham, Merck, UK).

#### 2.3.3 Transmission electron microscopy

The eye lavage EVs were processed for TEM as follows: EV pellets were resuspended in 100 mM sodium cacodylate buffer (pH 7.4). Approximately 3–5 μL of the EV suspension was then applied to a glow-discharged TEM grid with a carbon support film. The sample was partially air-dried for approximately 10 min before the grid was placed on a drop of fixative solution [2.5% glutaraldehyde (Agar Scientific Ltd, Stansted, UK) in 100 mM sodium cacodylate buffer, pH 7.4] for 1 min at room temperature. The grid was subsequently transferred across three drops of distilled water for washing, with excess water removed using filter paper. The grid was then placed on a drop of staining solution of 2% aqueous uranyl acetate (Agar Scientific Ltd, Stansted, UK) for 1 min, and any excess stain was removed with filter paper before air drying. TEM imaging of the EVs was conducted using a JEOL JEM 1400 microscope (JEOL, Tokyo, Japan) operated at 80 kV, with magnifications ranging from 30,000× to 60,000×. Digital images were captured using a GATAN Rio16 digital camera (Ametek GB Limited, Leicester, UK).

#### 2.3.4 Direct stochastic optical reconstruction microscope imaging of eye lavage EVs

The ONI EV profiler 2 kit and ONI Nanoimager system were used for labelling and visualisation of eye lavage EVs by direct stochastic optical reconstruction microscopy (dSTORM) (ONI UK, Oxford, UK). Following EV isolation, the EV samples were prepared according to the manufacturer's instructions (ONI, UK), following the manual sample preparation protocol (EV Profiler 2), using phosphatidylserine capture to capture the EVs on the chip surface, while EV visualisation was carried out by labelling with the following EV markers: tetraspanin trio [TT; anti-CD9 + CD63 + CD81 (561)] and a pan-EV marker (488). Imaging of EVs for Pan-EV and tetraspanin trio (TT) detection was carried out using the AutoEV imaging and CODI analysis protocol according to the manufacturer's specifications (ONI, UK).

### 2.4 Eye lavage EV proteome cargo analysis by LC-MS/MS and pathway enrichment analysis

EV proteome cargoes were analysed using LC-MS/MS following in-gel digestion (carried out by Cambridge Proteomics, Cambridge, UK). The EV preparations were run 0.5 cm into a 12% TGX gel and then cut out as one band for in-gel digestion. Protein hits were identified using the Mascot search algorithm (Matrix Science, London, UK) against the *Mus musculus* protein database (CC_Mus_musculus_20221011; 94,765 sequences; 39,873,048 residues). The ions score for hit identification was set at −10 × Log(*P*), where *P* was the probability that the observed match was a random event, with individual ions scores >31 indicating identity or extensive homology (*P* < 0.05). The fragment and peptide mass tolerances were set to 0.1 Da and 20 ppm, respectively.

For pathway enrichment analysis of the EV proteome cargoes, protein–protein interaction (PPI) networks were generated by uploading the protein hits into the STRING database (https://string-db.org/, accessed 25 March 2025) using the *Mus musculus* database with medium confidence settings. The PPI networks were compared using functional enrichment analysis in STRING, comparing the sham, naïve, and HI groups (non-affected and HI-affected eyes) and the shared and distinct Gene Ontology (GO), Reactome, and Kyoto Encyclopaedia of Genes and Genomes (KEGG) pathways relating to the proteins identified in the EV cargoes were found. The pathway analysis data of the protein networks were exported as STRING network images in the PNG format and as Excel files.

### 2.5 Statistical analysis

GraphPad Prism version 10 was used to compare the datasets from the different study groups. One-way ANOVA with the Bonferroni correction was used, with statistical significance at *p* ≤ 0.05. The STRING analysis was carried out with medium confidence (https://string-db.org/, accessed on 25 March 2025).

## 3 Results

**Figure 1:**
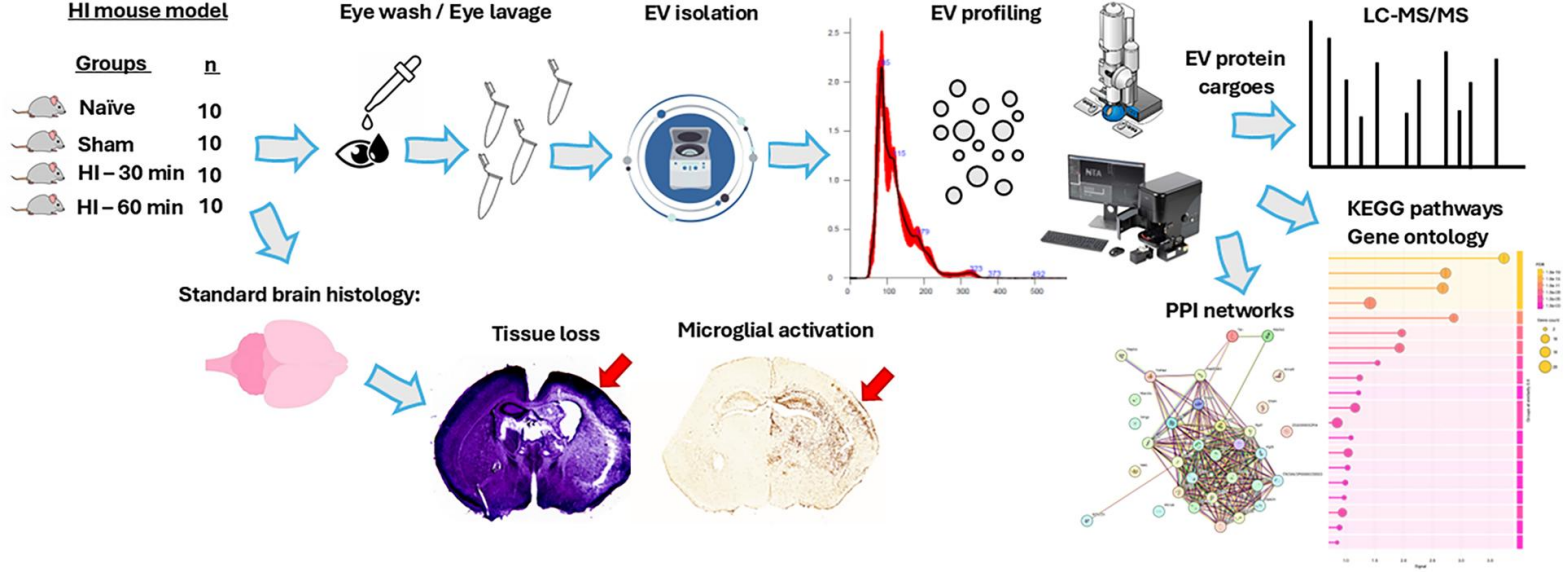
Schematic workflow. Eye lavage was collected from the eyes of naïve, sham, and HI animals. EVs were isolated by differential centrifugation, and EV profiling was carried out by NTA and microscopy, followed by EV protein cargo analysis by LC-MS/MS. EV proteomes were analysed for protein–protein interaction (PPI) networks and associated pathway enrichment (KEGG and GO pathways). HI damage was evaluated according to standard brain histology, with representative images shown for tissue volume loss (Nissl) and activated microglia (CD11b) in severe (60 min) HI at 48 h post-insult.

The level of HI insult was confirmed in both the mild (30 min) and severe (60 min) HI groups according to established methodologies, with validation of the model presented in previously published studies (7, 16, 17, 19) (Supplementary Figure S1). Figure 1 presents a schematic workflow for EV isolation and characterisation from eye lavage and downstream proteomic and network enrichment analyses, as well as brain sections representative of tissue volume loss (Nissl) and microglial activation (CD11b) at 48 h post-severe HI injury (Figure 1).

### 3.1 Characterisation and profiling of EVs from eye lavage

**Figure 2:**
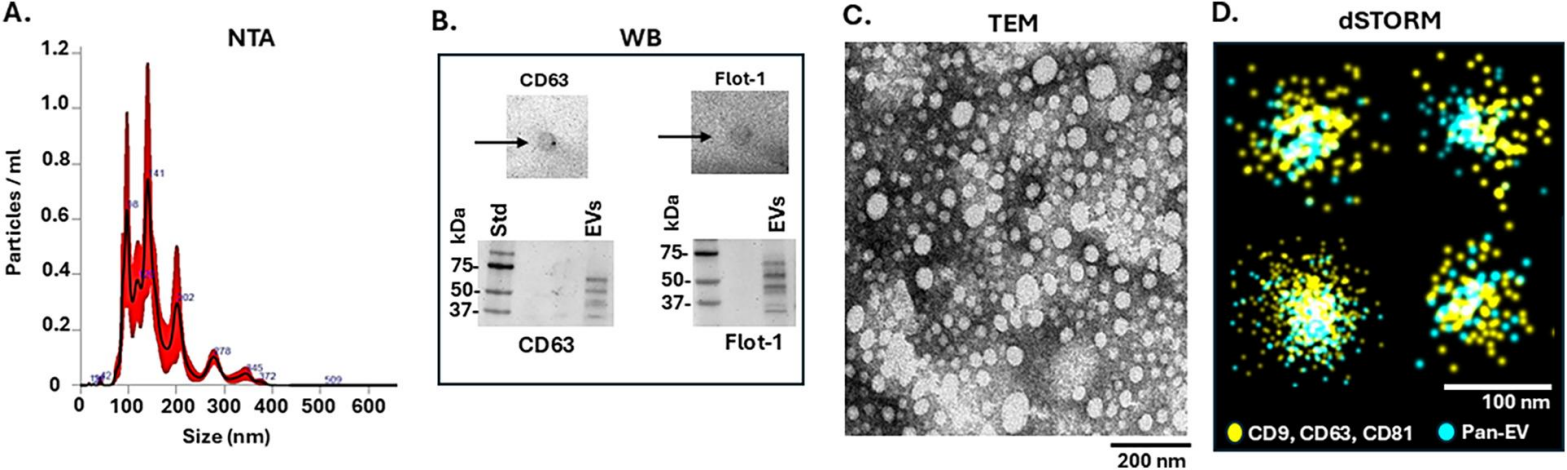
EV characterisation from eye lavage. **(A)** A representative nanoparticle tracking analysis (NTA) curve showing a poly-dispersed EV population in the size range of 40–350 nm. **(B)** Dot blotting and Western blotting (WB) are positive for two EV-specific surface markers: CD63 and Flot-1 (the size standard is indicated in kDa). **(C)** Transmission electron microscopy (TEM) image of eye lavage EVs (the scale bar indicates 200 nm). **(D)** dSTORM imaging of eye lavage EVs (yellow = tetraspanin trio CD9, CD63, CD81 marker; blue = pan-EV marker; scalebar = 100 nm).

EVs isolated from eye lavage were characterised according to recommendations by the International Society for Extracellular Vesicles on Minimal Information for Studies of Extracellular Vesicles (MISEV2023) (1). The NTA showed a polydisperse EV profile in the size range of approximately 40–350 nm, with main peaks observed around 90–145 nm (Figure 2A). The EVs were positive for two EV surface markers, CD63 and flotillin-1, in the dot blotting and WB (Figure 2B). The EVs were imaged by TEM (Figure 2C) and, in addition, positive detection of pan-EV and tetraspanin trio EV markers (CD9, CD63 and C81) (both from the ONI EV Profiler kit) was visualised at the single-molecule level using dSTORM imaging (Figure 2D).

#### 3.1.1 Changes in eye lavage EV numbers and size profiles following mild HI insult

**Figure 3:**
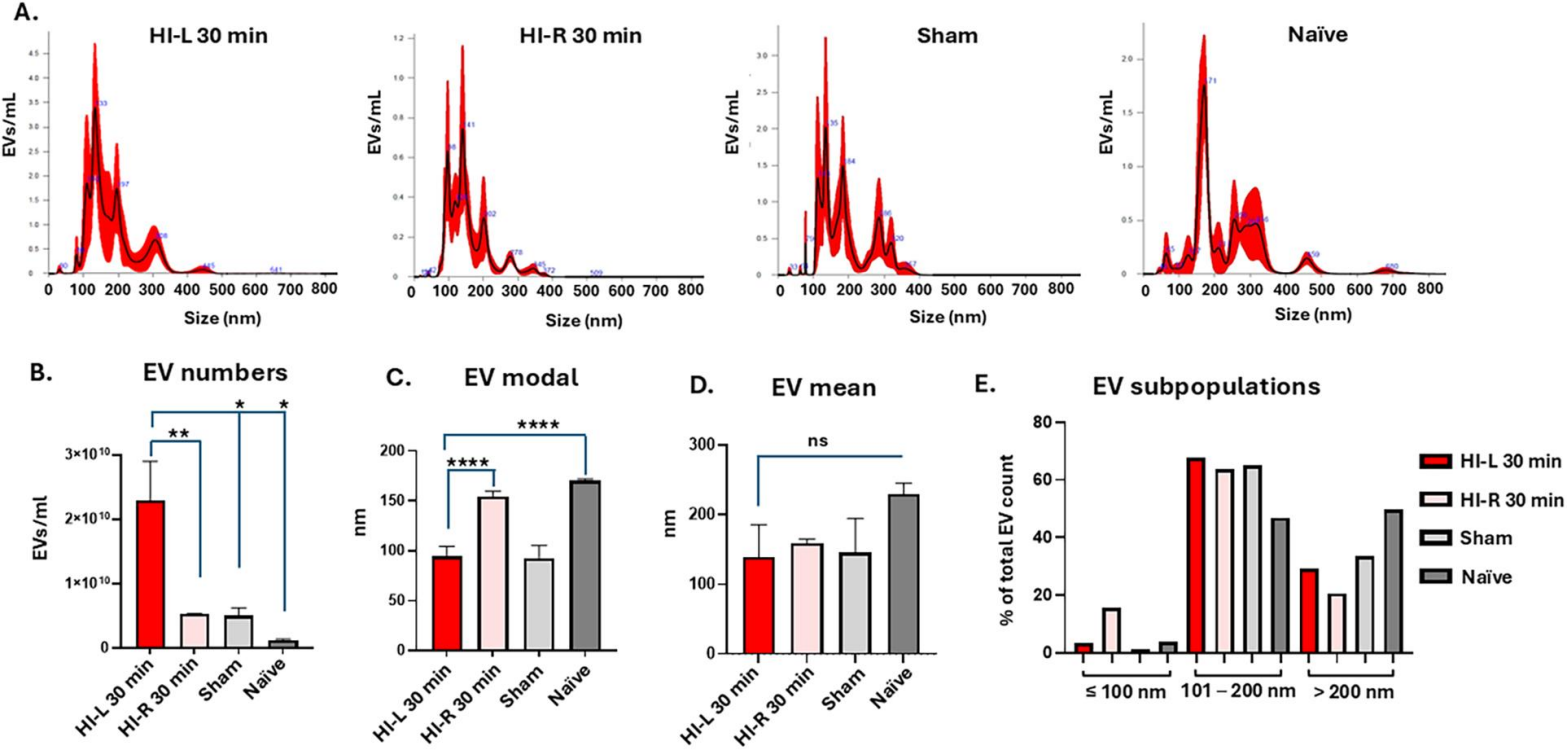
EVs from eye lavage were compared between the 30-min HI-affected animals and the sham and naïve controls. **(A)** Representative NTA curves from all four groups, showing the affected eyes (HI-L) in the 30-min HI treatment group, the unaffected eyes in the 30-min HI treatment group (HI-R, eyes on the non-occluded side) and sham and naïve groups. **(B)** EV eye lavage numbers isolated from the four groups show significantly higher numbers in the HI-L eyes compared with the other groups. **(C)** EV modal size was significantly smaller in the HI-L eyes compared with the eyes on the non-occluded side and the naïve eye group. **(D)** No statistically significant difference was observed in EV mean size when comparing the HI-affected eyes group with the other groups. **(E)** EV subpopulation analysis showed that the majority of EVs fell in the medium and large size range for all groups, and in the HI-affected eyes, there were more large EVs (>200 nm) than in the eyes on the non-occluded side, but fewer than in the sham and naïve controls (*n* = 10 eyes per group; error bars represent SD; *p < 0.05; **p < 0.01; ***p < 0.001; ****p < 0.0001; one-way ANOVA with the Bonferroni correction).

Changes in EV size distribution profiles and EV numbers were assessed between the sham, naïve, and HI groups, with the results for the 30-min HI group shown in Figure 3. Representative NTA curves for the 30-min HI experiment are shown in Figure 3A. Total EV numbers were significantly higher in the 30-min HI-affected eyes (HI-L) compared with the other groups (Figure 3B). The EV modal size analysis found significantly smaller EVs in the HI-L eyes compared to the non-affected (HI-R, i.e., the eye on the non-occluded side) and naïve eyes (Figure 3C). While the EV mean size was largest in the eye lavage of the naïve animals, no statistically significant differences were observed between the HI-L eyes and the other groups (Figure 3D). The EV subpopulation analysis, assessing small EVs (≤100 nm), medium-sized EVs (101–200 nm) and large EVs (>200 nm), showed that the majority of EVs fell in the medium and large EV size range in all groups, and, in the affected HI-L eyes, there were more large EVs (>200 nm) than in the non-affected HI-R eyes (on the non-occluded side), but fewer than in the sham and naïve controls (Figure 3E).

#### 3.1.2 Changes in eye lavage EV numbers and size profiles following severe HI insult

**Figure 4:**
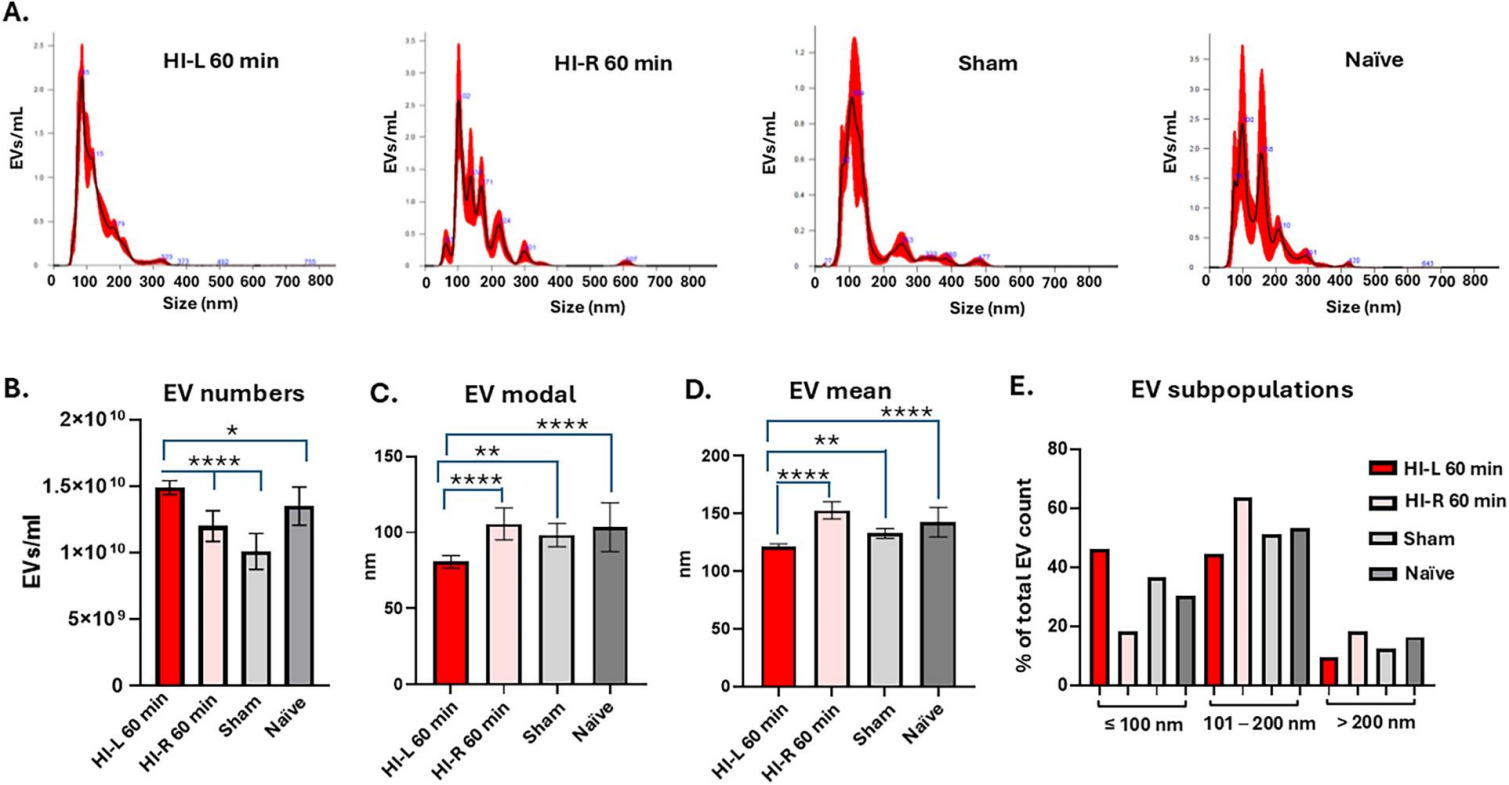
EVs from eye lavage were compared between the 60 min HI-affected animals and the sham and naïve controls. **(A)** Representative NTA curves from all four groups, showing the affected eyes (HI-L) in the 60-min HI treatment group, the unaffected eyes in the 60-min HI treatment group (HI-R, eye on the non-occluded side) and the sham and naïve groups. **(B)** EV eye lavage numbers isolated from the four groups show significantly higher numbers in the HI-L eyes at 60 min compared with the other three groups. **(C)** EV modal size was significantly smaller in the HI-L eyes compared with the other three groups. **(D)** EV mean size was significantly smaller in the affected HI eyes compared with the other three groups. **(E)** EV subpopulation analysis showing proportionally higher numbers of small and medium EVs. (*n* = 10 eyes per group; error bars represent SD; *p < 0.05; **p < 0.01; ***p < 0.001; ****p < 0.0001; one-way ANOVA with Bonferroni correction).

When performing the same analysis for eye lavage EVs from the 60-min HI conditions, significantly higher numbers of EVs were observed in eye lavage of the affected eye in the 60-min HI animals (HI-L), compared with the eye on the non-occluded side (HI-R) and the sham or naïve animals (Figure 4A). EV modal size was significantly smaller in eye lavage of the affected eye in the 60-min HI animals, compared with the HI-R (eyes on the non-occluded side) eyes and the sham or naïve animals (Figure 4C) and the same was observed for EV mean size (Figure 4D). When assessing EV sub-populations, the 60-min HI-affected eyes contained a higher proportion of small EVs (≤100 nm) but fewer medium EVs (101–200 nm) and large EVs (>200 nm), compared with the eyes on the non-occluded side and the sham and naïve groups (Figure 4E).

### 3.2 Proteome and functional enrichment pathway analysis of EV cargoes from eye lavage

**Figure 5:**
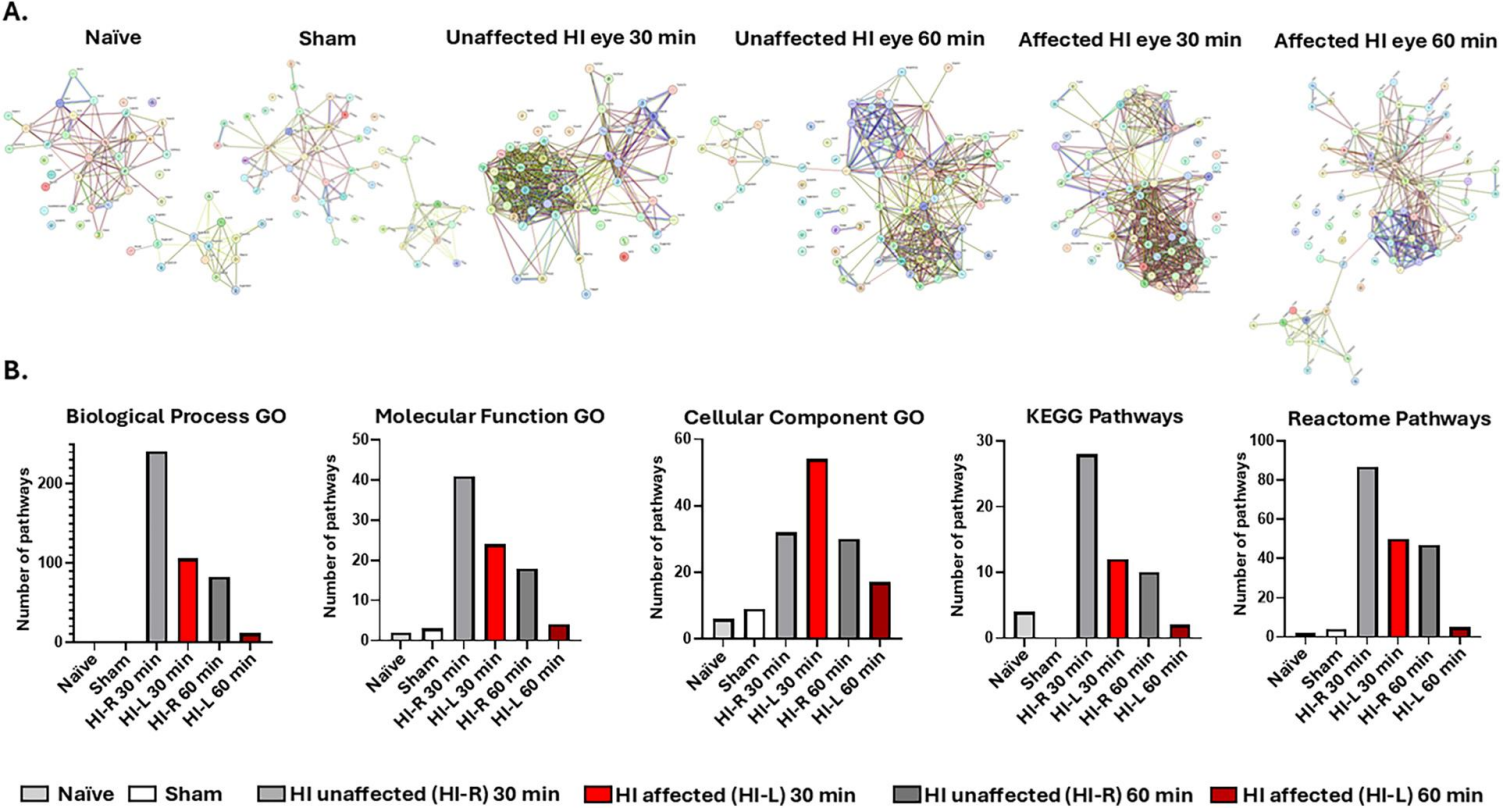
Protein–protein interaction (PPI) networks and functional pathway enrichment analysis for the proteomic cargoes of eye lavage EVs from naïve, sham, and HI (30 and 60 min) animals. **(A)** PPI networks for the EV eye lavage proteomic cargoes from the naïve, sham, and unaffected HI eyes (HI ctrl—HI-R, eye on the non-occluded side), and affected HI (HI-L) eyes in the 30 min and 60 min HI groups. **(B)** Functional enrichment pathway analysis of the EV proteomes from the presented experimental groups.

The eye lavage EV proteome cargoes from the control (sham and naïve) and experimental (30-min and 60-min HI) groups were analysed using LC-MS/MS and all the identified protein hits are summarised in Supplementary Table S1, showing the shared and unique protein hits among the groups. The proteins identified in eye lavage EVs were analysed for PPIs using STRING, creating PPI networks with each node presenting an identified protein (Figure 5A). A functional enrichment pathway analysis of the PPI networks was carried out to compare differences in the associated biological GO, molecular function GO, and cellular component GO pathways, and changes in the networks' associated KEGG and Reactome pathways in the control (naïve and sham) and HI (and 60 min) groups (Figure 5B).

#### 3.2.1 EV proteome signatures in eye lavage from naïve and sham animals

In the naïve group, one biological GO (intermediate filament organisation), two molecular function GO (s100 protein binding and structural molecule activity), and six cellular component GO pathways (cornified envelope, myelin sheath, intermediate filament, extracellular space, extracellular region, and cell cortex) were identified, while four KEGG pathways (oestrogen signalling pathway, *S. aureus* infection, *Salmonella* infection, and fluid shear stress and atherosclerosis) and two Reactome pathways (formation of the cornified envelope and developmental biology) were enriched for the eye lavage EV proteomes.

In the sham group, one biological process GO (intermediate filament organisation), three molecular function GO (antioxidant activity, s100 protein binding, and structural molecule activity), and nine cellular component GO (myelin sheath, collagen-containing ECM, extracellular space, extracellular region, cornified envelope, intermediate filament, dense body, keratin filament, and basement membrane) pathways were enriched. While no KEGG pathways were enriched, four Reactome pathways (keratinisation, formation of the cornified envelope, innate immune system, and developmental biology) were enriched for the eye lavage EV proteomes.

#### 3.2.2 EV proteome signatures in eye lavage of mild HI animals

In the HI groups, significantly more pathways were enriched for the eye lavage EV proteomes, both from the non-affected eyes (eye on the non-occluded side, hypoxia exposure only) and the affected eyes (hypoxia and ischaemia), compared with what was observed for the naïve and sham animals. It must be noted that while the unaffected eyes in the HI groups were not experimentally affected by ischaemic insult due to compensation of blood flow by the Circle of Willis, these eyes were still affected by hypoxia in the hypoxic chamber.

For the 30-min HI animals, the EV proteome of the non-affected eye (which was exposed to hypoxia only) was enriched for 214 biological GO, 41 molecular GO, 32 cellular GO, 28 KEGG, and 87 Reactome pathways. For the affected eyes of the 30-min HI animals (exposed to ischaemia and 30 min of hypoxia), the EV proteomes were enriched for 106 biological GO, 24 molecular GO, 54 cellular GO, 12 KEGG, and 50 Reactome pathways.

**Figure 6:**
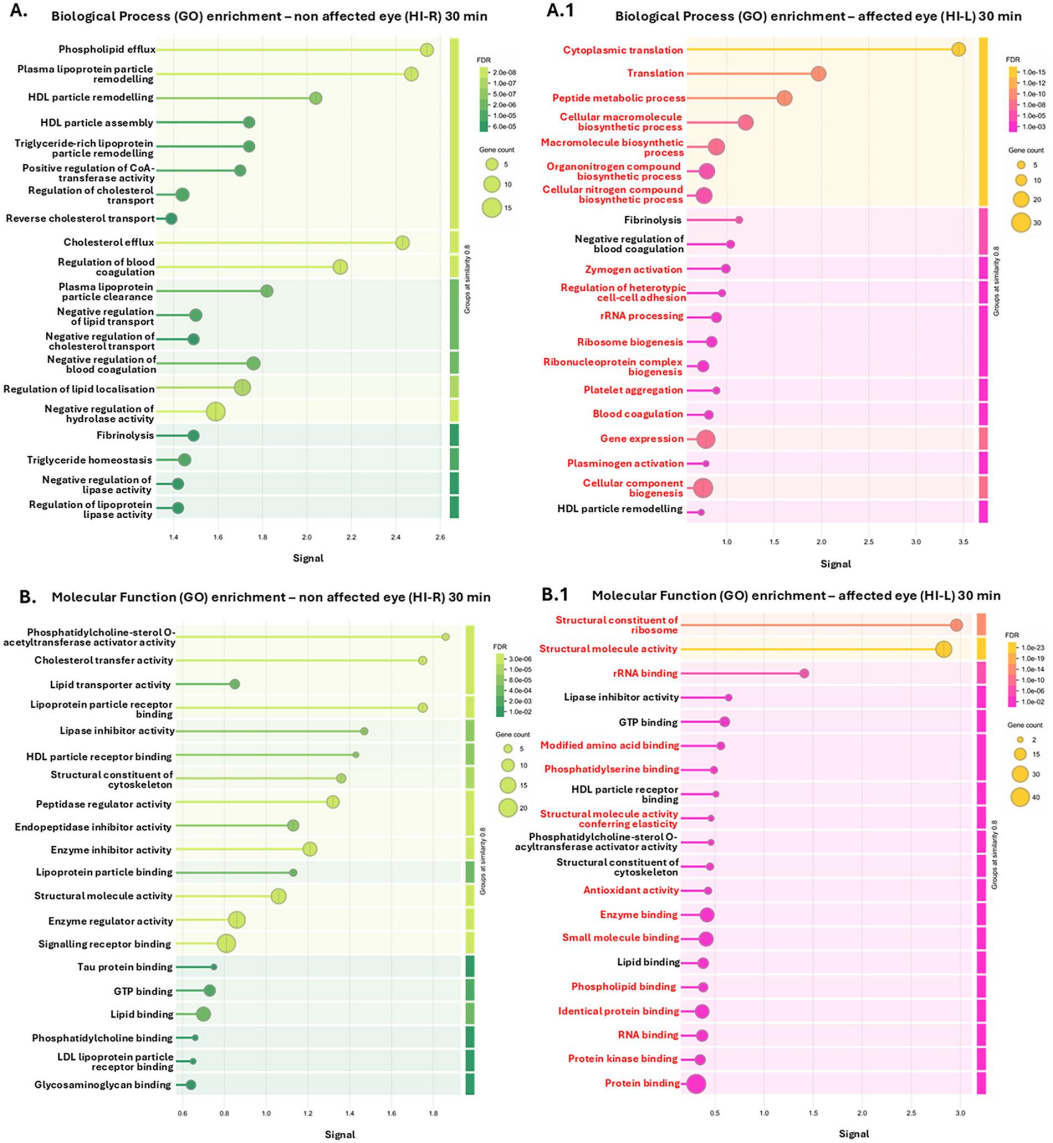
Pathway enrichment for the EV proteomes from eye lavage of non-affected HI eyes (HI-R, eyes on the non-occluded side and exposed to hypoxia only) in the 30-min HI animals **(A–E)** compared with their affected eyes (HI-L) **(A.1–E.1)**, comparing the top 20 enriched pathways based on signal and FDR. **(A,A.1)** Biological process GO pathways. (**B,B.1)** Molecular function GO pathways. **(C,C.1)** Cellular component GO pathways. **(D,D.1)** KEGG pathways. (**E,E.1)** Reactome pathways. The pathways that were only associated with the 30-min HI-affected eyes (**A.1–E.1**) (among the top enriched pathways shown) are highlighted in red.

The top 20 GO pathways, based on signal and false discovery rate (FDR), are shown for the eye lavage EV proteomes of the non-affected eyes (eye on the non-occluded side, exposed to hypoxia only) in the 30 min group in Figures 6A–E, and can be compared with those of the HI-affected eyes in the 30 min group in Figures 6A.1–E.1, respectively. The pathways that were only associated with the EV proteome of the 30-min HI-affected eye lavage (within the top enriched pathways shown) are furthermore highlighted in red.

#### 3.2.3 EV proteome signatures in eye lavage of severe HI animals

For the 60-min HI group, the eye lavage EV proteome of the non-affected eyes (eyes on the non-occluded side and exposed to hypoxia only) was enriched for 83 biological GO, 18 molecular function GO, 30 cellular component GO, 10 KEGG, and 47 Reactome pathways.

The eye lavage EV proteome of the 60-min HI-affected eyes (exposed to ischaemia and 60 min of hypoxia) was enriched for 12 biological GO, four molecular function GO, 17 cellular component GO, two KEGG, and five Reactome pathways.

**Figure 7:**
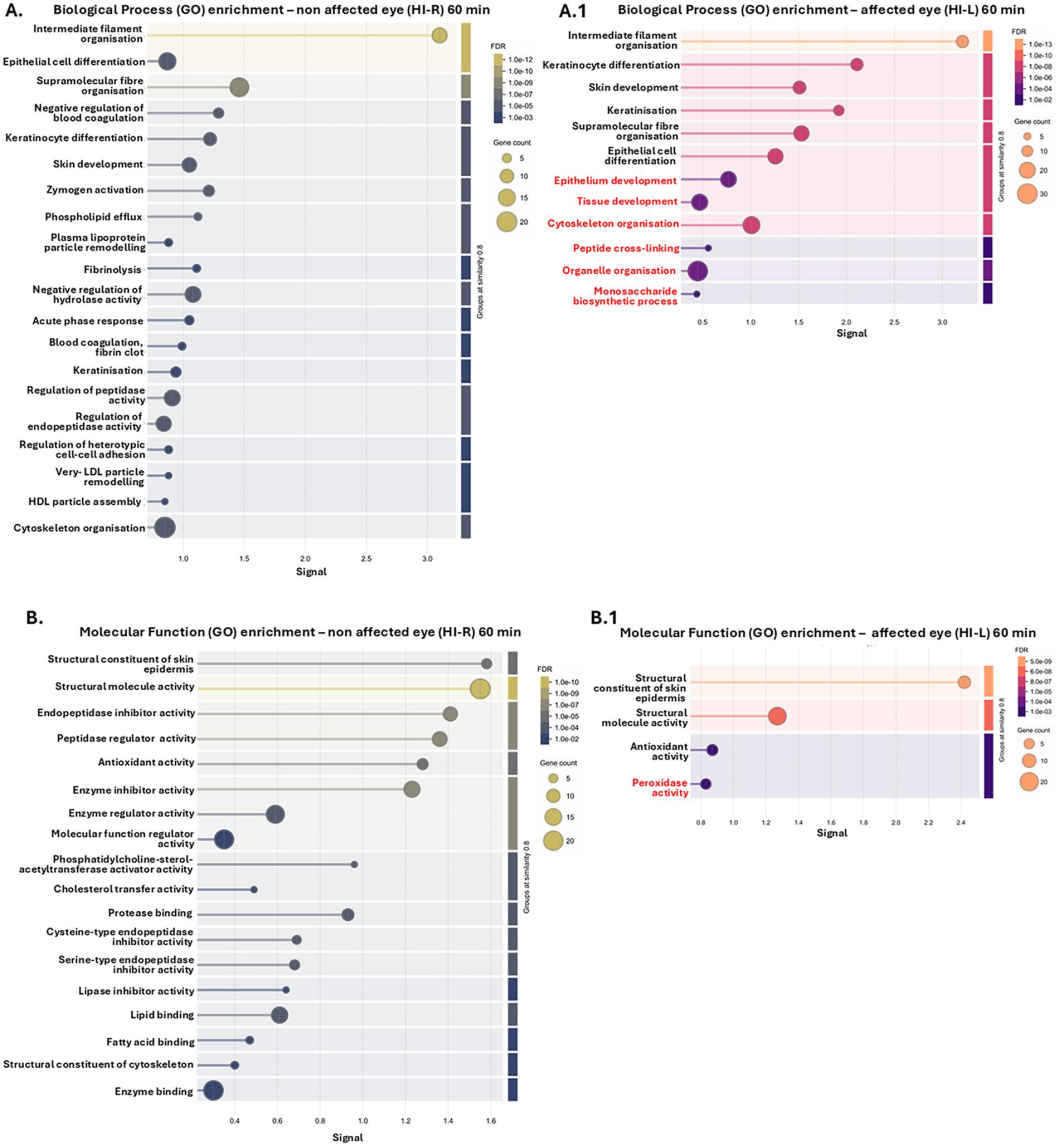
Pathway enrichment for the EV proteomes from eye lavage of non-affected eyes (eyes on the non-occluded side and exposed to hypoxia only, HI-R) in the 60-min HI animals **(A–E)** compared with their HI-affected (HI-L) eyes (**A.1–E.1**), comparing the top 20 enriched pathways based on signal and FDR. **(A,A.1)** Biological process GO pathways. **(B,B.1)** Molecular function GO pathways. **(C,C.1)** Cellular component GO pathways. **(D,D.1)** KEGG pathways. **(E,E.1)** Reactome pathways. The pathways that were only associated with the 60-min HI-affected eyes (**A.1–E.1**) within the top enriched pathways are highlighted in red.

The top 20 GO pathways, based on signal and FDR, are shown for the eye lavage EV proteomes of the non-affected HI eyes (exposed to hypoxia only) in the 60 min HI group in Figures 7A–E, and can be compared with those of the HI-affected eyes in the 60 min HI group in Figures 7A.1–E.1, respectively. The pathways that were only associated with the EV proteome from the 60-min HI-affected eyes (Figures 7A.1–E.1) are highlighted in red.

As a high number of proteins were specific to eye lavage EV cargoes from the affected eyes in the mild HI group, additional PPI network analysis and associated pathway enrichment were performed for these 31 protein hits. This excluded any shared hits with the other groups. This analysis showed 18 associated biological GO, five molecular function GO, 27 cellular GO, one KEGG (Ribosome), and 18 Reactome pathways. These results supported the findings from the whole EV proteome analysis in the 30 min HI group (shown in Figure 6) by showing enrichment in the GO, KEGG, and Reactome pathways linked to ribosome, RNA binding, translation, synapse, post-synaptic density, post-synapse, cell junction, RAP-1 signalling, and protein methylation (Supplementary Figure S2). When assessing the interactions and enrichment for the 21 unique protein hits identified in the 60-min HI-affected eye EVs, the PPI network for those specific hits showed enrichment for three pathways: protein heterotetramerisation, regulation of antibacterial peptide production, and mixed, including cornified envelope and desmosomal cadherin (not shown).

## 4 Discussion

This is the first study to isolate and characterise EVs from eye lavage in a neonatal model. This innovative non-invasive method may offer considerable clinical value in identifying biomarker signatures that reflect brain and eye injury following HI. Furthermore, this method may be translatable to other types of brain injury, neurodegenerative diseases, and eye pathologies.

Our study showed that EVs can be effectively isolated and utilised to characterise EV proteome signatures in very small sample amounts and from small sample sizes, as it was carried out in a neonatal mouse model at P11. This involved flushing the eyes with DPBS (20 µL per eye) and collecting the eye wash for EV isolation. This approach, therefore, shows promise for clinical translation, given the proportionally larger eye size of a human neonate compared with a P11 mouse pup, and should allow for individual analysis of human eye lavage. However, in this study, we were limited to pooling several samples per group due to the small eye size of the mouse pups.

The findings of this study revealed significant changes in eye lavage EV signatures in response to HI, using the neonatal mouse Rice–Vannucci model. EV numbers were significantly higher in the eye lavage of the HI-affected eyes compared with both the control non-affected HI eyes (on the non-occluded side) and the naïve and sham animals. This correlates with reports of changes in EV concentrations in biofluids such as plasma and cerebrospinal fluid (CSF) in a range of pathologies, including acute CNS injury (21–23). Importantly, proteome cargoes were considerably modified in the eye lavage EVs of the HI-affected animals in this study, and showed differing protein content and functional pathway enrichment in mild vs. severe HI.

### 4.1 Eye lavage EV protein cargoes associated only with mild HI and functional pathway enrichment

The protein hits that were only identified in the EVs of the mild (30 min) HI-affected eyes were associated with pathway enrichment for ribosome biogenesis, translation, RNA processing, gene expression, blood coagulation, innate immunity, antioxidant activity, phospholipid binding, post-synapse and post-synapse density, cell cortex, and HIF-1 signalling. The individual proteins that were only identified in the affected eyes of the 30-min HI group, and that did not overlap with proteins identified in other experimental groups, are briefly discussed below.

Heat shock protein 84b (part of the hsp90 class of proteins) and heat shock 70 kDa protein 8 (Hspa8) play roles in cellular homeostasis and are key players in cellular stress, including neuronal stress responses, with associations with neurodegenerative diseases (24–26). With respect to EVs, Hsp90 has regulatory roles in EV release (27), Hspa8 has been associated with multiple system atrophy and Parkinson's disease (28), and EV-mediated transport of heat shock proteins has roles in neuron-glia cross-talk during ageing (29).

Annexin 6 has roles in the secretion of small EVs and pro-inflammatory cytokines (30) and has also been shown to increase cancer aggressiveness via EV-mediated transport (31). Importantly, it has been reported as one of 12 key neuroinflammation markers (via gene expression profiling) that associate neonatal HI brain damage with progression to Alzheimer's disease (32).

Neuroblast differentiation-associated protein AHNAK is a nucleoprotein that has numerous roles in cellular responses, including calcium homeostasis (33) and EV production (34), and forms part of EV cargo in inflammatory diseases (35, 36). In the nervous system, roles for AHNAK are reported in Schwann cell myelination with putative implications for neuropathies (37), in spinal cord regeneration (38), and in neurological pathology associated with Huntington's disease (39). AHANK is also reported as a brain-associated marker for stress responses relating to parvalbumin-expressing interneurons, glutamatergic transmission, and post-synapse in chronic stress (40, 41), as well as a marker for age-related neurodegeneration (42).

Sarcoplasmic/endoplasmic reticulum calcium ATPase 1 (SERCA) is part of the calcium pump, with roles in the maintenance of cytosolic calcium levels and signalling (43). ATPase activity is associated with EV release, including in traumatic brain injury (44). SERCA pumps have roles in neurobiology, with associations with neuropathological conditions, including Parkinson's disease, Alzheimer's disease, and schizophrenia (45). It is also a reported marker of changes in spatial memory impairment due to neuronal apoptosis in the hippocampus (46).

Several ribosomal proteins were only identified in the eye lavage EVs of the 30-min HI-affected eyes. These are associated with various functions in translational control and inflammatory processes (47). The identified hits in this study were 40S ribosomal protein S3, related to glioma progression (48); 40S ribosomal protein S6, associated with neuronal function, including tau pathology (49, 50); 40S ribosomal protein S8; 40S ribosomal protein S25; 40S ribosomal protein S24; 60S ribosomal protein L6, which has been linked to metal-mediated pathogenesis in Parkinson's disease (51); 60S ribosomal protein L8, which has been associated with Diamond-Blackfan anaemia (52) and mTORC1 signalling (53); 60S ribosomal protein L9; 60S ribosomal protein L12; 60S ribosomal protein L14; 60S ribosomal protein L26, which has been associated to ischaemia-reperfusion injury (54); 60S ribosomal protein L27; 60S Ribosomal protein L32, which has reported roles in stress responses and cancer progression (55, 56); and 60S acidic ribosomal protein P2, which has reported roles in immune evasion (57) and is a marker of protein functions related to memory formation (58).

Isoform 6 of submandibular gland protein C (SMGC; Muc19 protein) was also unique to the eye lavage EVs of the 30-min HI animals. It has been identified as a putative Alzheimer's disease biomarker (59), is associated with Parkinson's disease (60), and is altered in ocular surface homeostasis in Sjögren syndrome (61).

Capping protein-inhibiting regulator of actin-like, also a unique hit, has roles in the promotion of actin polymerisation, with reported roles in neuroendocrine plasticity (62). It has also been studied in relation to phosphorylation in synapses and postsynaptic density (63, 64).

Elongation Factor 2 (Eef2) is essential for translational elongation and also has roles in the regulation of the overall rate of protein synthesis in neurons (65). Eef2-mediated protein synthesis has been reported to be modified by pH, which is relevant due to the tissue acidosis that accompanies hypoxia and ischaemia (66). Furthermore, it has been associated with neurodevelopmental disorders linked to developmental delay, hydrocephalus, autism, and Down syndrome, and with adult-onset spinocerebellar ataxia type 26 (SCA26) and Alzheimer's disease (67–72).

Synaptic vesicle membrane protein VAT-1 homolog (VAT1) is a component of synaptic vesicles involved in neurotransmitter transport and was originally reported in the electric organs of marine rays, with human homologues identified (73, 74). It has roles in mitochondrial fusion, phospholipid transport, and cell migration and is present in various organs during zebrafish development, including the neural tube, gut, brain, and retina (75, 76).

T-complex protein 1 has roles in protein folding and quality control within cells, including in relation to misfolded proteins associated with neurodegeneration (77). Dysregulation of T-complex protein 1 has been reported in mesial temporal lobe epilepsy (78). It is associated with Down syndrome, as studies have pointed to its significant contribution to neuropathogenesis in the foetal cerebral cortex (79). Furthermore, it is associated with normal retinal development and mutations in the gene (*cct2*) encoding for the protein that has been associated with retinal pathology (Leber congenital amaurosis; LCA), a rare, inherited genetic eye disease causing severe vision loss from birth or early infancy (80).

Rap1 (Ras-related protein RAP-1A) is a small GTPase protein involved in regulating cell adhesion and proliferation. It affects calcium and inflammatory pathways and regulates signalling pathways affecting integrins and cadherins (81). Rap1 is associated with various neuronal functions, including neuronal cell polarity, synaptic transmission and plasticity, dendritic spine formation, and neurite outgrowth (82–84). Roles for Rap1a have been identified in cerebral ischaemia injury (85), cerebral and spinal cord cavernomas (86), multiple sclerosis (87), and refractory epilepsy (88). With respect to neurodevelopmental disorders, Rap1a has been associated with autism spectrum disorder (89). Changes in Rap1 signalling have also been implicated in the development of preeclampsia (90) and in eye and lens development with relevance for associated pathologies, including macular degeneration (91, 92).

80K protein/MARCKS (myristoylated alanine-rich C-kinase substrate) is an actin filament cross-linking protein with roles in cell signalling pathways, cell motility, membrane trafficking, mitogenesis, and phagocytosis. It is associated with brain development and plasticity, neuronal and glial cells, and the regulation of ependymal barrier function (93–97). MARCKS deficiency has been related to abnormal brain development and perinatal death in mice (98) and it has been shown to have roles in neural progenitor cell dynamics in brain development (99). MARCKS has been suggested as a therapeutic target to promote axon regeneration after CNS injury, including in spinal cord and optic nerve regeneration (100, 101), and as having roles in ischaemic stroke (102, 103). MARCKS has been investigated in the ageing brain, has been associated with frontotemporal lobar degeneration, and has been highlighted as a sex-specific biomarker for Alzheimer's disease (104–106).

EH-domain containing 4-KJR (EHD4) was also only identified in the 30-min HI eye EVs. It has important roles in endocytic membrane trafficking (107), including in neurones and axonal transport and polarisation (108, 109). EDH4 has roles in synaptic plasticity, with roles in early postnatal brain development, and has been associated with post-stroke damage in the ageing brain (110). It has also been identified as a marker associated with tau neuropathology in progressive supranuclear palsy (111).

14-3-3 protein zeta/delta belongs to the 14-3-3 protein family and is abundantly expressed in the brain and enriched at synapses, with roles in synaptic function (112, 113). It is reported as a cerebrospinal fluid biomarker of synaptic dysfunction in several neurodegenerative diseases and is strongly associated with Alzheimer's disease (114–117). Furthermore, it is associated with neurodevelopmental and neuropsychiatric behaviour defects (118, 119). Roles in neuronal migration, neuronal progenitor cells, neuronal crest cell development, and pigmentation defects have also been reported (120–122). Changes in 14-3-3 proteins are a marker of glutamate-induced neonatal neuronal damage (123) and have been identified as markers of sex-specific brain morphology and behaviour in a model of neonatal rat exposure to estradiol-17β (124).

Histone H2B has key roles in DNA packaging and gene regulation, with roles in transcriptional activation and elongation. In ischaemic stroke, histone H2B has been reported to have roles in neural reprogramming (125), oxygen-induced retinopathy (126), and HI, including with respect to therapeutic targeting (127). Studies on epigenetic effects in ocular disorders have focused on several histones, including H2B (128) and histone modifications for therapeutic targeting (129).

Titin is a key muscle-associated protein linked to various muscular diseases, including dystrophy (130). Forms of titin have also been identified in neurones in the central and peripheral nervous systems, localised to the nucleolus at the site of ribosomal RNA biogenesis, with implicated roles in neurodegenerative disease (131). Titin autoantibodies have been reported as cerebrospinal fluid biomarkers in psychiatric autoimmune encephalitis (132) and in psychotic symptoms linked to inflammation or ischaemic injury (133).

It is of considerable interest that the eye lavage EVs showed such marked changes in mild HI, indicating that EV signatures can be further developed as reliable biomarkers for the effect of mild HI, where it is often difficult to identify that an insult has occurred, in addition to predicting any downstream and/or longer-term outcomes. It must be noted that EVs were collected 48 h post-injury in this study, as this is the timepoint at which we routinely assess histological changes, including microglial activation, cell death markers, and brain tissue volume loss (7, 16, 17, 19). At this timepoint post mild HI insult, an elevation in microglial activation, alongside some TUNEL positive staining, indicative of neuronal cell death, was detected in the hippocampus (Supplementary Figure S1), while significant tissue volume loss was not observed (Supplementary Figure S1). Several of the proteins specific to eye lavage EVs from the affected 30-min HI eyes are indeed linked to neurological and eye conditions, as discussed above, and coincide with the mechanisms underlying neonatal HI pathology and the observed neuroinflammatory responses and associated cell death in the HI-affected brains at this timepoint. In future in-depth studies, the selected EV proteome candidates should be further evaluated regarding their correlation with specific brain tissue and inflammatory markers, and with respect to a wider time window post-mild-HI-insult.

### 4.2 Eye lavage EV protein cargoes associated with severe HI only and functional pathway enrichment

The enriched pathways that were only associated with the EV proteome of the severe HI group were related to cytoskeleton organisation, peptide cross-linking, monosaccharide biosynthesis, peroxidase activity, extrinsic component of plasma membrane, GAIT complex, mast cell granulation, innate immune system, ruffle, and sealing of the nuclear envelope by the endosomal sorting complex required for transport III (ESCRT-III). The protein hits that were only identified in the eye lavage EVs from the severe (60 min) HI animals and that did not overlap with proteins identified in the other experimental groups are briefly discussed below.

Annexin 1 (ANXA1), a member of the Annexin superfamily, is a calcium-dependent binding protein with various functions in the nervous system, including as an essential mediator in blood–brain barrier (BBB) integrity, and diverse roles in neuroinflammatory responses (134). It has roles in microglial activation and microglia/macrophage polarisation in acute ischaemic stroke and traumatic brain injury (135–137). ANXA1 has been shown to reduce BBB permeability in sepsis-associated encephalopathy (138), while in neonatal HIE, depletion of ANXA1 is associated with temporal loss of BBB integrity and is suggested as a therapeutic target (139). In the eye, ANXA1 is found in retinal Müller cells and microglia, with upregulation in activated microglia (140, 141). ANXA1 is associated with various eye diseases, including glaucoma and retinopathy due to hypoxia and ischaemia (134, 142–144).

Keratin, type I cytoskeletal 17 (Krt17) forms part of the cell cytoskeleton and is a multifunctional protein with roles in cell growth, migration, proliferation, signal transduction, and apoptosis (145). In the brain, it has been identified to be altered in the hippocampus and striatum in a chronic alcohol neurotoxicity mouse model (146). In the eye, Krt17 has been reported to have important roles in the cornea, including in relation to the stemness, proliferation, and differentiation of limbal stem cells, and in immune defence responses (147).

Deleted in malignant brain tumours 1 protein (DMBT1) is a glycoprotein with roles in innate immunity, angiogenesis, and epithelial differentiation. In amniotic fluid, it correlates with gestational age (148) and has been associated with several neonatal pathologies, including hypoxia and gastrointestinal and pulmonary diseases. Its expression is upregulated in several neonatal gastrointestinal diseases in preterm and term infants, such as volvulus, intestinal perforation, herniation, and necrotising enterocolitis, and in cardiac anomalies and sepsis (149, 150). In the lung tissue of neonates, DMTB1 is upregulated in response to respiratory stress and in lung epithelial cell models exposed to hypoxia (151). DMBT1 isoforms have previously been reported in human tears (152) and are associated with dry eye syndrome (153) and conjunctival melanoma (154).

Secretion-associated Ras-related GTPase 1A (SAR1a) is a small GTPase with roles in the vesicle-mediated endoplasmic reticulum (ER) to Golgi transport pathway and protein export. In glioblastoma, it is linked to the regulation of apoptosis and redox homeostasis (155) and has also been linked to autophagy (156). SAR1a also has roles in lipid homeostasis, cell protection against oxidative stress, and inflammatory processes (157). Furthermore, SAR1a was identified as a potential biomarker in Alzheimer's disease, with differential expression in human brains with Alzheimer's disease (158).

Calmodulin 4 is a calcium-binding protein with regulatory roles in cellular functions, including wound healing (159). Calmodulin antagonists have been identified to have neuroprotective roles in various animal models of focal cerebral ischaemia and hypoxia-ischaemia (160–162). Furthermore, calmodulin 4 has been identified as an addiction-related gene that is elevated in the prefrontal mouse cortex (163). It was identified to have regulatory roles in hypoxia-induced autophagy (164) and is involved in pregnancy-dependent adaptations to hypoxia (165). In rats, calmodulin 4 upregulation has been associated with corneal epithelial and nerve regeneration via the cAMP signalling pathway (166).

Tubulin polymerisation-promoting protein family member 3 (TPPP3) is a microtubule-associated protein with roles in tubulin polymerisation and stabilisation of microtubules (167). It is critical for myelination (112) and is associated with oligodendrogliopathy (168). In relation to neurodegeneration, TPPP3 was shown to counteract alpha-synuclein aggregation in Parkinson's disease pathology (169) and it is implicated in diabetic neuropathy (170). In fish models, TPPP3 was shown to play roles in axon regeneration (171). Furthermore, it forms part of the glial component of the optic nerve (172) and was recently highlighted as a novel molecule for retinal ganglion cell identification in mice, macaques, and humans. Moreover, it has been found to have roles in promoting neuroinflammatory pathways and genes involved in optic nerve regeneration (173).

Tubulin beta-4A chain (TUBB4a) belongs to the β-tubulin gene family, with critical roles in microtubule polymerisation, axonal transport, myelination, and neuronal migration (174–176). It is developmentally regulated, with high expression in the foetal brain, including the cortex and cerebellum. Pathogenic variants of TUBB4A are implicated in paediatric-onset leukodystrophies, affecting myelin development (177, 178). Mutations in TUBB4a have been associated with hypomyelination, including cerebellar atrophy (179), hereditary dystonia (180), hereditary spastic paraplegia (181, 182), and epileptic encephalopathy associated with hypomyelinated leukodystrophy (183).

Bifunctional glutamate/proline–tRNA ligase (Eprs1) is a multifunctional protein with roles in immunity and metabolism, including via peptide chain elongation, rRNA aminoacetylation, and the mTORC1 signalling pathway (184, 185). It has roles in mediating inflammatory homeostasis by regulating AKT signalling, with implications in sepsis and chronic inflammation (186). EPRS1 is associated with hypomyelinated leukodystrophy (187, 188) and psychomotor developmental delay, epilepsy, and deafness (189).

Vitamin K-dependent gamma-carboxylase (BVKGC) is responsible for the post-translational modification of glutamate residues to gamma-carboxy glutamic acid and affects a wide range of physiological pathways relating to haemostasis and signal transduction (190). It plays roles in calcium flux and metabolic stress adaptation (191), blood coagulation (192), and bone calcification, and is related to bone and metabolic disorders (193). Furthermore, BVKGC has been associated with rare bleeding and coagulation disorders (194, 195). Roles for vitamin K in preventing oxidative damage to developing neurons and oligodendrocytes have been suggested, with possible applications in perinatal HI brain injury (196).

Transferrin has multifaceted roles, including in iron homeostasis. In the brain, oxidative stress and ferroptosis can contribute to brain injury and are implicated in various neurological conditions, including neurodegeneration and neonatal brain injury (197–199). Iron homeostasis is also critical for oligodendrocyte development, with changes related to hypomyelination and neurological impairments (200). HI has been shown to affect the spatiotemporal expression of iron-related proteins in neurons, blood vessels, and oligodendrocytes in neonatal rat brains (201). In a longitudinal study, increased transferrin levels and reduced ferritin levels in the CSF were associated with improved neurodevelopmental outcomes in neonatal post-haemorrhagic hydrocephalus (202). In neonatal HI, maternal and supplemented lactoferrin have been shown to be neuroprotective, including by preventing mitochondrial and redox homeostasis dysfunction (203, 204). In a preclinical rat study of retinopathy of prematurity, neonatal anaemia was associated with worse outcomes in oxygen-induced retinopathy, with more adverse outcomes for females (205).

Truncated profilaggrin/filaggrin flaky tail mutant form, also identified in the 60-min HI-affected eye lavage EVs, is associated with a defective skin barrier and various skin conditions, including ichthyosis and dermatitis (206). It has been identified as a single-nucleotide polymorphism (SNP) associated with developmental profiles of latent classes of allergic diseases including eczema, atopic dermatitis and asthma (207).

4-Hydroxy-2-oxoglutarate aldolase mitochondrial (Hoga1) is a mitochondrial enzyme with critical roles in hydroxyproline metabolism and in generating pyruvate and glyoxylate. It is associated with hyperoxaluria type-3, which often presents in childhood and is characterised by recurring calcium oxalate stones (208–210). In a recent genome-wide association study (GWAS), it was furthermore identified as a risk factor in Alzheimer's disease (211).

Loricrin is a major structural protein in the cornified envelope, providing a barrier against external stressors, and is involved in crosstalk between keratinocytes and epidermal resident leukocytes (212). Mutations in the loricrin gene are linked to several congenital skin abnormalities, including various forms of ichthyosis (213). It is expressed in all mammalian stratified epithelia *in vivo*, with the highest levels of expression in newborn epidermis, oral and anal mucosal epithelium, oesophagus, vagina and foreskin, and epidermal parts of the sweat ducts (214). Its expression in the stratified squamous corneal epithelium is associated with corneal injury, including dystrophy formation and scarring (215), as well as in the conjunctiva in severe ocular surface disease (216).

F-box only protein 50 (FBX50), or non-specific cytotoxic cell receptor protein 1 (NCCRP1), is involved in the regulation of the cell cycle, cell proliferation, signal transduction, and ubiquitin protein ligase activity (217). It is associated with various cancers, including as an ER stress-related gene, with roles in proliferation, migration, invasion, and apoptosis (218). In chronic inflammation, it has been highlighted as a potential stress-response biomarker (219) and it is involved in the cytotoxic cell-mediated immune response and regulation of signalling pathways and apoptosis in teleost fish (220, 221).

Dihydropyrimidinase (Dpys) is a key enzyme in the pyrimidine synthesis pathway and is implicated in various cancers (222, 223). Dpys deficiency has been related to neurological and gastrointestinal abnormalities (224). Dpys is associated with various paediatric neurometabolic phenotypes (225). Several genetic Dpys-related pathologies include infantile spasm, seizure disorder, reduced or abnormal white matter, brain atrophy, and delay in speech development (226–228). Downregulation of Dpys has been reported to be protective in sepsis encephalopathy (229).

Charged multivesicular body protein 4b (CHMP4B) forms part of the ESCRT-III machinery with multifaceted roles, including in membrane repair and apoptosis (230). Mutations in the gene are related to early-onset lens cataracts and lens opacities, while it is required for lens growth and differentiation, including via gap junction protein complexes during lens fibre cell differentiation (231, 232). In traumatic brain injury, its upregulation was shown to reduce microglial necroptosis and neuroinflammation (233). Roles for CHMP4B in the autophagy-lysosomal pathway in Alzheimer's disease have also been reported (234).

Peroxiredoxin-5 (Prdx5) belongs to a large superfamily of thioredoxin peroxidases. Prdx5 acts as an antioxidant and is localised to peroxisomes, cytosol, nuclei, and mitochondria, with strong expression in neuronal mitochondria (235). Pdrx5 was found to be a critical target for reducing oxidative stress and cell apoptosis in early brain injury in a severe rat stroke model of subarachnoid haemorrhage (236). It is also suggested as a serum immune-response biomarker for ischaemic stroke diagnosis (237). Pdrx5 was shown to have roles in modulating neuronal apoptosis in acute spinal cord injury (238). Neuroprotective roles, i.e., scavenging peroxides, have been reported in ischaemia-reperfusion injury (239). In neurodegenerative diseases, Prdx5 has been linked to changes in redox homeostasis (240) and to increased iron overload-induced neuronal death (241). Prdx5 has been reported in the retina and optic nerve and may be of relevance in various oxidative stress-related eye pathologies (242).

SURF6 domain-containing protein is a component of the nucleolar matrix and is essential for ribosome biogenesis, rRNA processing, and cell proliferation (243). It is associated with activated lymphocytes in lymphocytic leukaemia and is a suggested biomarker for human blood disorders (244). SURF6 has been shown to be essential for mouse preimplantation development via the regulation of ribosome biogenesis (245). GWASs have identified SURF6 in a causal association between chronic heart failure and the cerebral cortex (246) and between gut microbiota and white matter injury and connectivity (247).

Arginase I (Arg1) is a key part of the urea cycle, which is important for metabolic aspects of neuronal pathologies, including neurodegenerative diseases and acute CNS injury (248, 249). Arg1 plays important roles in macrophage phenotypic characteristics and is a canonical marker for M2 anti-inflammatory macrophages in relation to tissue repair, including in CNS injury (250–253). Arg1 has, for example, been associated with improved outcomes in cerebral ischemia (254, 255) and spinal cord injury (256). Arg1 has been identified in MSC-EVs, promoting microglial M2 polarisation after subarachnoid haemorrhage in rats (257). Interestingly, in a recent study on ischaemic stroke, Arg1 was reported to have unexpected detrimental influences on infiltrating macrophages by modulating microglial function and synaptic pruning in the peri-infarct region, affecting post-stroke recovery (258). Arg1 may, therefore, be a spatial-temporal marker for CNS injuries and in relation to therapeutic time windows for targeting immune cells in the brain (259). In relation to brain cancer, EVs from tumour-associated macrophages were shown to contain Arg1 and promote glioblastoma progression (260). In the eye, Arg1 has been shown to be upregulated in optic nerve injury (261). Furthermore, it has been found to have anti-inflammatory microglial function in retinal degeneration (262) and immunoregulatory roles in retinal Müller glial cells in autoimmune uveitis (263).

N6-adenosine-methyltransferase subunit (Mettl3) is involved in mRNA methylation, mRNA regulation, and translation control. It regulates cell proliferation and is implicated in cancer and metabolic and cardiovascular diseases (264). Mettl3 is a key regulatory protein that is associated with nervous system development, with roles in regulating neurophysiological events (neurogenesis and glial and synaptic plasticity), and is implicated in neuropathological events, including autism, neurodegenerative disorders, and brain injuries (265, 266). Roles for Mettl3 have been reported in intracerebral haemorrhage and ischaemic stroke (267, 268), including neonatal HIE, where Mettl3 was shown to contribute to microglial activation and inhibit microglial pyroptosis (265, 269). Furthermore, roles in regulating autophagy via mTOR signalling have been reported in cerebral ischemia-reperfusion injury (270). In the eye, Mettl3 affects the planar cell polarity of lens epithelial cells, with implications in myopic cataract formation (271) and oxidative stress (272). Upregulated Mettl3 has been associated with corneal endothelial dystrophy (273), while the inhibition of Mettl3 is implicated in the regulation of retinal ganglion cell ferroptosis in glaucoma (274). Roles for Mettl3 in retinal development have been reported, including the maintenance of retinal photoreceptor function (275).

Similarly to the 30-min HI group, EVs from the 60-min HI group were collected at 48 h post-injury, the timepoint at which the brain histology assessment showed significant changes (at a higher level than in the mild HI group) in microglial activation, as indicated by strong CD11b immunoreactivity (Supplementary Figure S1). Moreover, high levels of cell death, as reflected by strong TUNEL-positive staining (Supplementary Figure S1), particularly in the hippocampus and in other brain regions, and significant tissue volume loss in several brain regions, which was particularly significant in the cortex and hippocampus (Supplementary Figure S1 and Figure 1), were found. Many protein hits unique to the severe HI-affected eye lavage EVs identified here and discussed above are involved in the pathological mechanisms of neonatal HI damage. They indicate an association with the neuronal function, neuroinflammatory, and degenerative pathways and are related to brain and eye-related pathologies. It is, therefore, important to prioritise the candidates identified here as specific to the severe HI group for further assessment, validation, and analysis of their correlation with brain and eye tissue changes, including at different time points post-injury. This will also be important for establishing time windows for diagnosis and differentiation between mild and severe neonatal HI insult.

This study focused specifically on EV isolation from eye lavage, rather than assessing total eye lavage for proteomic profile differences, as the EV proteome cargo is diluted in whole eye lavage assessment. Furthermore, our approach may be transferable as it uses purified EVs, which can be isolated from various eye lavage volumes, resulting in enriched EV pellets that can then be further processed. As previously mentioned, while we pooled eye lavage from several mouse pups for EV isolation due to their small eye size, it must be considered that human neonatal eyes are larger, and one would expect a larger EV yield per sample. Our protocol described herein should therefore be applicable both for smaller pooled samples and for larger volumes of individual eye lavage samples, providing value for potential clinical translation.

In summary, the comparison of EV cargo protein hits in eye lavage revealed notable differences between the mild (30 min) and severe (60 min) HI groups with respect to the affected eyes exposed to hypoxia and ischaemia. While there were links to numerous neuroinflammatory and neuropathological functions in both HI groups, the mild HI group had more associations with ribosome-associated functions, including translation, RNA processing, and gene expression. In addition, the mild HI group had associations with HIF signalling, antioxidant activity, and phospholipid binding. Furthermore, various proteins were associated with synaptic function, including post-synaptic signalling. In the severe HI group, the eye lavage EV cargo proteins were associated with cytoskeleton organisation, peptide cross-linking, monosaccharide biosynthesis, peroxidase activity, extrinsic component of plasma membrane, GAIT complex, mast cell granulation, ruffle, and sealing of the nuclear envelope by ESCRT-III.

The use of EVs as biomarkers is widely studied in a range of human pathologies, while research in neonatal HI is still limited. In a HI mouse model, plasma EVs have shown some promise for selected markers, but no sex-specific differences were detected (276). In a group of near-term/term neonates with HIE, neuronal proteins in serum EVs have been reported as putative biomarkers for predicting responses to therapeutic hypothermia treatment (277). Further investigations into the use of EVs as biomarkers in HI are therefore of high importance. Our reported findings in this study highlight the use of ocular fluid EV-based biomarkers and describe a new non-invasive method using eye lavage EVs, which may be of considerable value in clinical applications. The selected protein candidates for mild vs. severe HI found in our initial proteomic analysis will need to be further validated in future studies with respect to time windows post-injury, differentiation between acute and chronic responses, and sex-specific differences. While the current study used a neonatal HI mouse model, these findings can form the basis for further research and development of sophisticated and translatable non-invasive EV platforms for clinical use.

## 5 Conclusion

This study developed a new non-invasive method using eye lavage EVs to identify changes in response to neonatal hypoxic ischaemic brain insult. Our results highlight that eye lavage EV signatures can be used as non-invasive biomarkers, with the potential to predict changes that occur in the brain and eye due to different severities of HI injury. EVs were elevated in eye lavage from HI-affected animals. Comparing the EV proteomes of mild vs. severe HI revealed significant differences in target proteins and the enrichment of associated GO and KEGG pathways relating to various inflammatory, stress, and disease pathways. Future studies should focus on confirming the selected protein candidates for mild vs. severe HI with respect to a wider range of time windows and sex-specific differences. The development of eye lavage EV biomarker platforms has translational potential for diagnostic applications in a range of other neurological and eye-related pathologies.

## Data Availability

The original contributions presented in the study are included in the article/Supplementary Material, further inquiries can be directed to the corresponding authors.
