# Supplementary material for: Extracellular vesicle signatures from eye lavage as novel non-invasive biomarkers for hypoxic ischaemic insult—findings from a neonatal mouse model
Source: Front Med Technol. 2025 Dec 18;7:1715676. doi: 10.3389/fmedt.2025.1715676 (PMC12756510; doi:10.3389/fmedt.2025.1715676)
Supplement: Supplementary file 1 [file Datasheet1.pdf]

Supplementary Material

Extracellular Vesicle Signatures from Eye Lavage as Novel Non-invasive Biomarkers for Hypoxic Ischaemic Insult – Findings from a Neonatal Mouse Model

Runci Li[1], Sarah R. Needham[2], Igor Kraev[3], Mariya Hristova[1]*, Sigrun Lange[1,4]*

[1] Department of Neonatology, EGA Institute for Women's Health, University College London, London WC1E 6BT, U.K.

[2] UKRI: Science & Technology Facilities Council, Central Laser Facility, Rutherford Appleton Laboratory, Oxfordshire, OX11 0QX, U.K.

[3]Electron Microscopy Suite, Faculty of Science, Technology, Engineering and Mathematics, Open University, Milton Keynes MK7 6AA, U.K.

[4]Pathobiology and Extracellular Vesicles Research Group, School of Life Sciences, University of Westminster, London W1W 6UW, U.K.

*** Correspondence:**
Mariya Hristova m.hristova@ucl.ac.uk and Sigrun Lange s.lange@westminster.ac.uk

1.1 Supplementary Figures

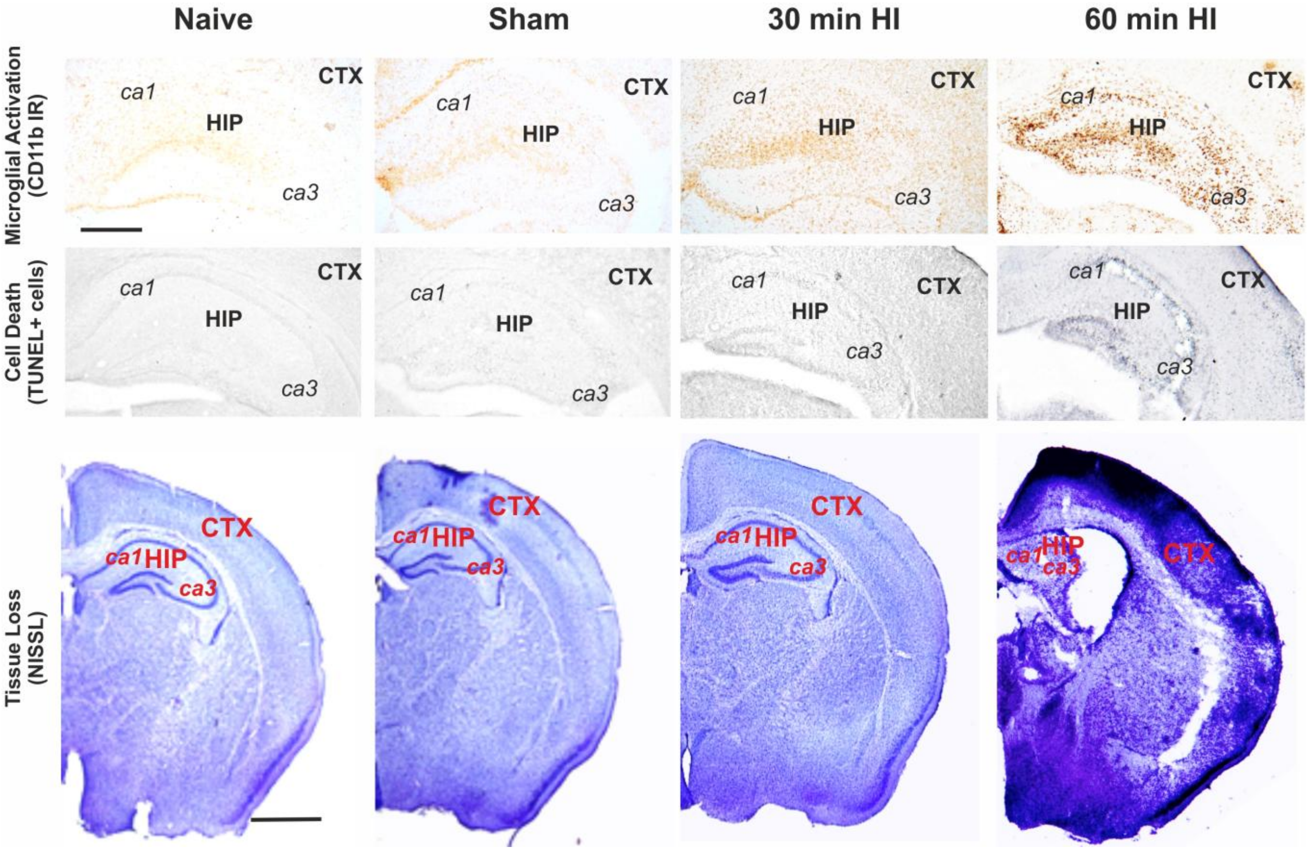

Supplementary Figure 1. Brain tissue histology of naïve, sham operated, mild (30 min) and severe (60 min) HI P11 mouse brains, 48 h post insult. Microglial activation is shown in hippocampus (HIP) and cortex (CTX) through CD11b immunoreactivity. Note the considerably stronger staining in the severe (60 min) HI brains, while positive staining is also observed in the mild (30 min) HI brains compared to naïve and sham controls. Cell death is assessed through terminal deoxynucleotidyl transferase-mediated dUTP nick-end labeling assay (TUNEL), with increased number of TUNEL+ cells reflective of higher levels of cell death in the severe (60 min), compared with the mild (30 min) HI brains, as well as the naïve and sham controls. Tissue volume loss assessed through cresyl violet (NISSL) staining shows significant loss of brain tissue in the severe (60 min) HI brains, while tissue loss is not significantly marked in the mild (30 min) HI brains at 48 h post insult, and not present in naïve or sham operated control animals. Hippocampal areas ca1 and ca3 are also indicated in the images. Scale bars: microglial activation and cell death =1000 μm, tissue loss =500 μm.

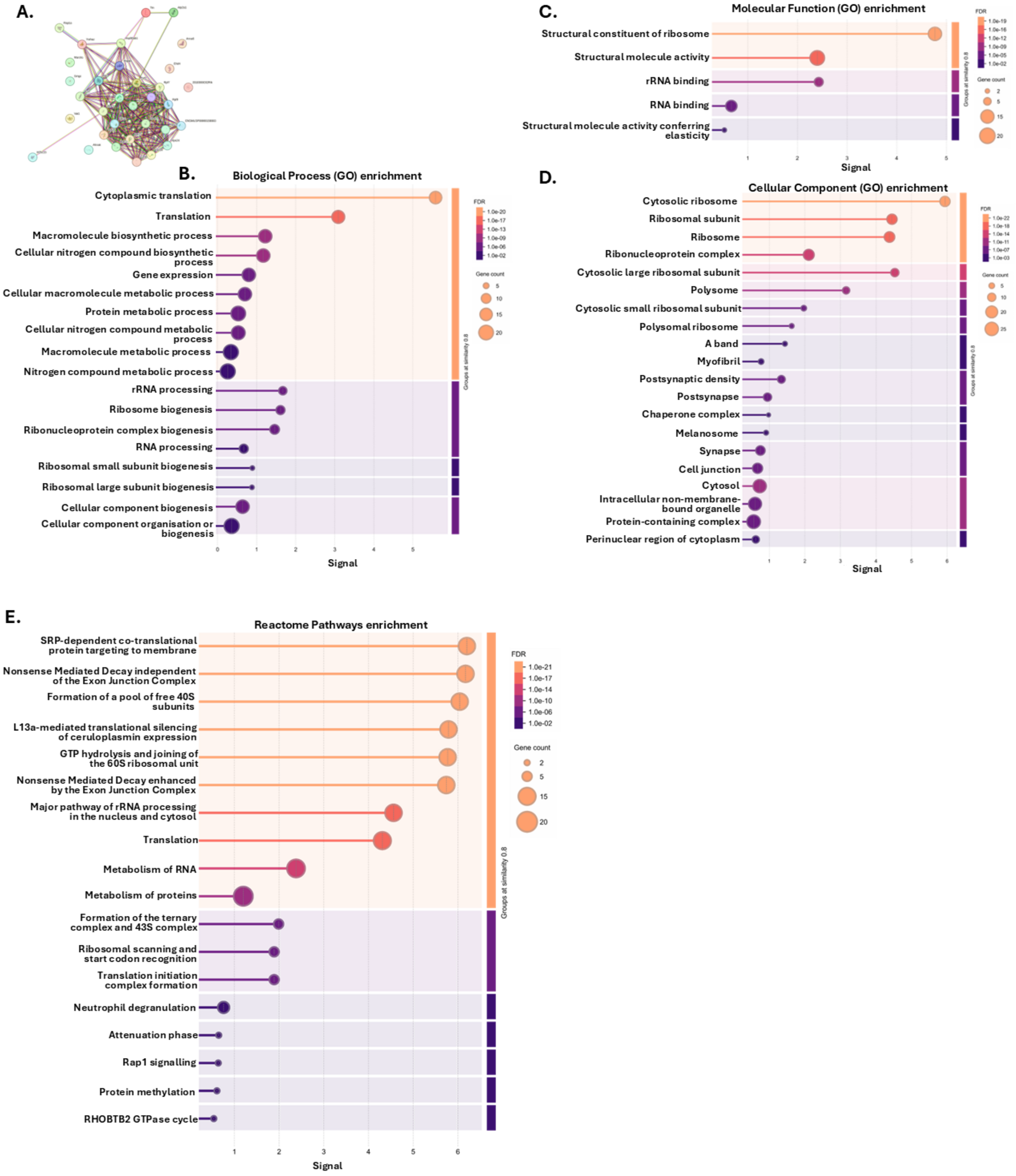

Supplementary Figure 2. EV proteome hits specific only to the affected HI 30 min eye. PPI and Pathway enrichment analysis. A. PPI network. B. Biological Process GO. C. Molecular Function GO. D. Cellular Component GO. E. Reactome pathways.

Supplementary Table

Supplementary Table 1. LC-MS/MS analysis of EV protein cargoes from eye lavage. Protein hits identified in the EVs of each group are listed and a tick (V) indicates that they are present in the respective group. EVs isolated from eye lavage were compared from naïve, sham, unaffected eye of 30 min HI (HI-R), affected eye of 30 min HI (HI-L, unaffected eye of 60 min HI and affected eye of 60 min HI; n= 10 eyes per group.

Protein ID	Protein Name	Naïve	Sham	HI – R 30 Unaffected Eye 30 min	HI – L 30 Affected Eye 30 min	HI-R 60 Unaffected Eye 60 min	HI-L 60 Affected Eye 60 min
P97384 ANX11	Annexin A11	V					
A1L317 K1C24 Krt24	Keratin, type I cytoskeletal 24	V					
Q148R7 Hrnr	Hrnr protein	V					
J3QM75 Scgb2b19	ABPBG19	V					
G3UXN8 Scgb1b27	Secretoglobin, family 1B, member 27	V					
D3Z4A4 Prdx2	Peroxiredoxin-2	V					
Q9D6P8 CALL3	Calmodulin-like protein 3	V					
A0A1L1SRM3 Rbpms2	RNA-binding protein with multiple-splicing 2	V					
Q3U9U3 Tubb6	Tubulin beta chain	V	V				
Q9D3I0	RIKEN cDNA 5530400C23 gene	V	V				
A2ANT5 Mup4	Major urinary protein 1	V	V				
A8DUV3 Hbat1	Alpha-globin		V				
Q3TL58 Skp1	S-phase kinase-associated protein 1		V				
T1ECW4 Rbpms	RNA-binding protein with multiple-splicing		V				
Q3TFK4 Anxa3	Annexin		V				
A2A591	Keratin-associated protein 3-1		V				
Q9Z2T6 KRT85	Keratin, type II cuticular Hb5		V				
Q07456 AMBP	Protein AMBP			V			
Q3TJD4 Atp5pb	ATP synthase subunit b			V			
A2A5L3 Ncoa5	Nuclear receptor coactivator 5			V			
Q549A5 Clu	Clusterin			V			
B2RSN3 Tubb2b	Tubulin beta chain			V			
P05213 TBA1B Tuba1b	Tubulin alpha-1B chain			V			
P56480 ATPB	ATP synthase subunit beta, mitochondrial			V			
A7YL62	Apolipoprotein A-II			V			

Apoa2							
A0PJ91 Hsp90aa1	Hsp90aa1 protein			V			
Q3TR40 Thbs1	Uncharacterised			V			
Q3TAN1	Clathrin-link domain-containing protein			V			
B1ATS4 Atp2a3	Calcium-transporting ATPase			V			
Q61268 APOC4	Apolipoprotein C-IV			V			
P29788 VTNC	Vitronectin			V			
Q3V1T9 Plg	Plasminogen			V			
E9Q414 APOB	Apolipoprotein B-100			V			
Q8BVI9 Slc25a4	ADP/ATP translocase			V			
Q3TIZ0 Tuba1c	Tubulin alpha chain			V	V		
P01027 CO3	Complement C3			V	V		
A0A0R4J1N3 Apoc3	Apolipoprotein C-III			V	V		
P99024 TBB5	Tubulin beta-5 chain			V	V		
Q71LX8 Hsp90ab1	Heat shock protein 84b				V		
Q99JX6 Anxa6	Annexin6				V		
Q6UL10 Ahnak	AHNAK				V		
Q3KQJ4 Hspa8	Hspa8 protein				V		
A0A1L1SUF6 Rpl14	60S ribosomal protein L14				V		
Q8R429 Atp2a1	Sarcoplasmic/endoplasmic reticulum calcium ATPase 1				V		
Q3UBI6 Rpl7	Uncharacterized protein, 40S ribosomal protein S3				V		
Q6JHY2-5 SMGC	Isoform 6 of Submandibular gland protein C				V		
Q3TIQ2 Rpl12	60S ribosomal protein L12				V		
Q3UCH0 Rpl6	60S ribosomal protein L6				V		
A0A286YEB7	40S ribosomal protein S24				V		
E9Q3M9 Cracdl	Capping protein-inhibiting regulator of actin-like				V		
Q3TJZ1 Eef2	Tr-type G domain-containing Protein; Elongation Factor 2				V		
A0A0G2JES3 Rpl9	60S ribosomal protein L9				V		
Q3THC7 Rpl8	60S ribosomal protein L8				V		
Q3TXD3 Vat1	PKS_ER domain-containing Protein				V		
Q9JJD8 Cct2	T-complex protein 1 subunit beta				V		
A0A0G2JDL9 Rap1a	Ras-related protein Rap-1A				V		
A0A1L1SQA8 Rps25	40S ribosomal protein S25				V		

A0A0A1HAM8 Marcks	80K protein				V		
A0JLV3 Hist1h2bj	Histone H2B				V		
Q3TL53 Rps6	40S ribosomal protein S6				V		
B1ARA3 Rpl26	60S ribosomal protein L26				V		
Q5PR09 Rpl32	Ribosomal protein L32				V		
Q5BLJ9 Rpl27	60S ribosomal protein L27				V		
A0A140LI77 Rps3	40S ribosomal protein S3				V		
A0A5F8MPY2 Rplp2-ps1	60S acidic ribosomal protein P2				V		
Q5M9L9 Rps8	40S ribosomal protein S8				V		
Q1MWP8 Ehd4	EH-domain containing 4-KJR				V		
A0A2I3BQ03 Ywhaz	14-3-3 protein zeta/delta				V		
A0A5K1VVQ1 Ttn	Titin				V		
A0A0A6YW67 Gm8797	Predicted pseudogene 8797				V		V
Q3TE63 Ppia	Peptidyl-prolyl cis-trans isomerase				V		V
Q91X72 Hpx	Hemopexin					V	
Q3UD36 Vim	Vimentin					V	
K1C28 Krt28	Keratin, type I cytoskeletal 28					V	
Q3TGA3 Afp	Uncharacterized protein					V	
Q3U9Q8 Gsn	Gelsolin					V	
A0A0A0MQA5 Tuba4a	Tubulin alpha chain					V	
Q3V2G3 Prss3	Peptidase S1 domain-containing protein					V	
Q3TFG3 Eif4a1	RNA helicase					V	
Q0VGU8 Bpifa6	BPI fold-containing family A, member 6					V	
G3X977 Itih2	Inter-alpha-trypsin inhibitor heavy chain H2					V	
A2AJD1 Bpifb9b	BPI fold-containing family B, member 9B					V	
A0A0U1RPN8 Aldoa	Fructose-bisphosphate aldolase					V	
P11087-2 CO1A1 Col1a1	Isoform 2 of Collagen alpha-1(I) chain					V	
A0A1D5RM76 Tubb3	Tubulin beta-3 chain					V	
A0A087WPA9 Scgb2b18	ABPBG18					V	
D6REU3 Cstdc5	Cystatin domain-containing 5					V	
Q0VDR7 Krt6b	Krt6b protein					V	V

P07744<br>Krt4	Keratin, type II cytoskeletal 4					V	V
P70124<br>Serpinb5	Serpin B5					V	V
Q3TCL2<br>Akr1b3	Aldo_ket_red domain-containing protein					V	V
Q3TNN6<br>D3YTL5<br>Capg	Macrophage-capping protein					V	V
Q9DCV7<br>Krt7	Keratin, type II cytoskeletal 7					V	V
Q8CGL5<br>Cstdc6	Stefin A1					V	V
P24472<br>Gsta4	Glutathione S-transferase A4					V	V
Q7M745<br>Scgb2b26	Allergen dI chain C2D					V	V
Q3TAS8<br>Dpysl3	Amidohydro-rel domain-containing protein					V	V
Q3UU48<br>Pip	Prolactin-inducible protein homolog					V	V
A0A494BBD8<br>Anxa1	Annexin-1						V
Q9QWL7<br>Krt17	Keratin, type I cytoskeletal 17						V
A0A140LHB2<br>Dmbt1	Deleted in malignant brain tumors 1 protein						V
A0A1W2P6N3<br>Sar1a	GTP-binding protein SAR1a						V
Q6WEH7<br>Calmodulin 4	Calmodulin 4						V
Q1JPR8<br>Tppp3	Tubulin polymerization-promoting protein family member 3						V
Q8CGC7<br>Eprs1	Bifunctional glutamate/proline--tRNA ligase						V
B2RS80<br>Ggcx	Vitamin K-dependent gamma-carboxylase						V
E9Q2Q7<br>Trf	Transferrin						V
TBB4A<br>Tubb4a	Tubulin beta-4A chain						V
C1KG51<br>Flg	Truncated profilaggrin/filaggrin flaky tail mutant form						V
Q9DCU9<br>Hoga1	4-hydroxy-2-oxoglutarate aldolase, mitochondrial						V
P18165<br>Loricrin	Loricrin						V
A0A0A0MQ97<br>Nccrp1	F-box only protein 50						V
Q8K0X3<br>Dpys	Dpys protein						V
Q9D8B3<br>Chmp4b	Charged multivesicular body<br>protein 4b						V
A0A494BAZ4<br>Prdx5	Peroxiredoxin-5						V
Q3V1X4<br>Surf6	SURF6 domain-containing protein						V
Q8K3K5	Arginase I						V
A0A0R4J041<br>Mettl3	N6-adenosine-methyltransferase subunit METTL3						V
Q3UYE3	Uncharacterized						V

Gm5570							
B2RRX1<br>Actb	Actin, beta	V	V	V	V	V	V
Q3TII3<br>Q3UA81<br>Eef1a1	Elongation factor 1-alpha	V	V	V	V	V	V
Q32P04<br>Krt5	Keratin 5	V	V	V	V	V	V
A0A0A0MQF6<br>Gapdh	Glyceraldehyde-3-<br>phosphate dehydrogenase	V	V	V	V	V	V
Q3U344<br>Arf3	ADP-ribosylation factor	V	V	V	V	V	V
D2XZ37<br>Scgb2b2	ABPBG2	V	V	V	V	V	V
A0A0A6YVU7<br>Flg	Filaggrin	V	V	V	V	V	V
Q61781<br>K1C14	Keratin, type I cytoskeletal<br>14	V		V	V	V	V
B0V2N5<br>Anxa2	Annexin	V	V		V	V	V
Q66VB7<br>Gm1553	Lacrein	V	V		V	V	V
Q545F4<br>D3YZ06<br>Hspb1	Heat shock protein beta-1	V	V		V	V	V
A0A089N3E7<br>Abpbg15_bg17	ABPBG15_bg17	V	V		V	V	V
F6WYC8<br>Lipo1	Lipase	V	V		V	V	V
Q9D3H2<br>OBP1A	Odorant-binding protein 1a	V	V		V	V	V
A2AEP0<br>OBP1B	Odorant-binding protein 1b	V	V			V	V
A1E2B8	Inducible heat shock<br>protein<br>70	V	V			V	V
A0A8D3UE05<br>Asprv1	Retroviral-like<br>aspartic protease 1	V	V			V	V
A8R0V1<br>Esp18	Exocrine gland secreted<br>peptide 18	V	V			V	V
A2AEN9<br>Gm5938	Predicted gene 5938	V	V			V	V
Q5SW46<br>PERL	Lactoperoxidase	V	V			V	V
O09131<br>A0A494B9X6<br>GSTO1	Glutathione S-transferase<br>omega-1	V	V			V	V
G3X9V8<br>Serpinb3a	Serine (or cysteine)<br>peptidase<br>inhibitor, clade B<br>(ovalbumin), member 3A	V	V			V	V
Q61782	Type I epidermal keratin<br>mRNA,<br>3'end	V	V	V	V		V
B1AXW5<br>Prdx1	Peroxiredoxin-1	V	V		V		V
P50543<br>S10AB	Protein S100-A11	V	V		V		
Q545I9<br>S100a6	Protein S100	V	V				V
D3YYY1<br>Scgb2b7	ABPBG7	V	V				V
D2XZ31<br>Scgb1b29	ABPA7	V	V				V

A0A3B2WD20 Esp6	Exocrine gland secreted peptide 6	V	V				V
A0A2I3BRY2 Mucl2	Mucin-like protein 2	V	V				V
Q7M754 Gm5409	Try10-like trypsinogen	V	V	V			V
Q5FW97 EG433182	Phosphopyruvate hydratase	V		V	V	V	V
Q3TLH6 Fabp5	FABP domain-containing protein	V		V	V		V
B1AQ78 Krt19	Keratin 19	V			V	V	V
P04104 K2C1	Keratin, type II cytoskeletal 2	V				V	V
P08730 Krt13	Keratin, type I cytoskeletal 13	V				V	V
J3QK77 Scgb2b20	ABPBG20	V				V	V
A0A0A6YWJ1 A0A0A6YXF6 Rhoa	Transforming protein RhoA	V					V
G3UYV7 Rps28	40S ribosomal protein S28	V					V
P50446 K2C6A	Keratin, type II cytoskeletal 6A		V		V	V	V
Q8BGZ7 K2C75	Keratin, type II cytoskeletal 75		V		V		V
Q792Z1 Try10	Trypsin 10		V	V	V	V	V
A8DUK2 A8DUK4 Hbbt1	Beta-globin		V	V	V	V	V
Q546G4 Alb	Serum albumin		V	V	V	V	V
Q9Z1R9 Prss1	Protease, serine 1 (trypsin 1)		V	V	V	V	V
B1AQ77	Keratin, type I cytoskeletal 15		V	V		V	V
Q3TGR2 Fgb	Fibrinogen beta chain			V	V	V	V
Q8BPF4	GLOBIN domain-containing protein			V	V	V	V
P68372 TBB4B	Tubulin beta-4B chain			V			V
Q3UKA4 Adh1	PKS_ER domain-containing protein		V				V
Q6IFX2 K1C42	Keratin, type I cytoskeletal 42	V	V	V		V	
Q3U7V7 Pfn1	Profilin	V		V	V	V	
A2AQL0 Stk39	Non-specific serine/threonine protein kinase	V	V	V	V		
A0A0N4SV85 Pip	Prolactin-induced Protein, Pip		V			V	
A1A535-2 Veph1	Ventricular zone-expressed PH domain-containing protein 1		V			V	
Q921I1 TRFE	Serotransferrin		V	V	V	V	
Q3UEM7	Fibrinogen C-terminal domain-containing protein		V	V	V	V	
Q3UBS3	Haptoglobin		V		V	V	
B2RTM0	Histone H4		V		V		

Hist2h4							
E9PV24-2<br>FIBA<br>Fga	Isoform 2 of Fibrinogen<br>alpha<br>chain			V	V	V	
A0A0R4J038<br>Kng1	Kininogen-1			V	V	V	
Q9DBN0<br>Apoa4	uncharacterised			V	V	V	
Q3V2G1<br>Apoa1	uncharacterised			V	V	V	
Q3UBP6<br>Actb	uncharacterised			V	V	V	
A0A087WR50<br>Fn1	Fibronectin			V		V	
Q3TIU3<br>A0A338P703<br>Ahsg	Alpha-2-HS-glycoprotein			V		V	
A0A2K6EDJ7<br>Itih4	Inter alpha-trypsin<br>inhibitor, heavy chain 4			V		V	
Q61838<br>PZP	Pregnancy zone protein			V			
A0A0A0MQA3<br>Serpina1a	Alpha-1-antitrypsin<br>1-1			V		V	
Q3TX45<br>Apoe	Uncharacterised			V		V	
P34928<br>APOC1	Apolipoprotein C-I			V		V	
